# PIM kinases regulate early human Th17 cell differentiation

**DOI:** 10.1016/j.celrep.2023.113469

**Published:** 2023-11-30

**Authors:** Tanja Buchacher, Ankitha Shetty, Saara A. Koskela, Johannes Smolander, Riina Kaukonen, António G.G. Sousa, Sini Junttila, Asta Laiho, Olof Rundquist, Tapio Lönnberg, Alexander Marson, Omid Rasool, Laura L. Elo, Riitta Lahesmaa

**Affiliations:** 1 Turku Bioscience Centre, University of Turku and Åbo Akademi University, 20520 Turku, Finland; 2 InFLAMES Research Flagship Center, University of Turku, 20520 Turku, Finland; 3 Department of Microbiology and Immunology, University of California San Francisco, San Francisco, CA 94143, USA; 4 Institute of Biomedicine, University of Turku, 20520 Turku, Finland; 5 Gladstone-UCSF Institute of Genomic Immunology, San Francisco, CA 94158, USA; 6 Department of Medicine, University of California San Francisco, San Francisco, CA 94143, USA

**Keywords:** PIM kinases, T helper cell differentiation, Th1 cells, Th17 cells, transcriptomics, bulk RNA sequencing, single-cell RNA sequencing

## Abstract

The serine/threonine-specific Moloney murine leukemia virus (PIM) kinase family (i.e., PIM1, PIM2, and PIM3) has been extensively studied in tumorigenesis. PIM kinases are downstream of several cytokine signaling pathways that drive immune-mediated diseases. Uncontrolled T helper 17 (Th17) cell activation has been associated with the pathogenesis of autoimmunity. However, the detailed molecular function of PIMs in human Th17 cell regulation has yet to be studied. In the present study, we comprehensively investigated how the three PIMs simultaneously alter transcriptional gene regulation during early human Th17 cell differentiation. By combining PIM triple knockdown with bulk and scRNA-seq approaches, we found that PIM deficiency promotes the early expression of key Th17-related genes while suppressing Th1-lineage genes. Further, PIMs modulate Th cell signaling, potentially via STAT1 and STAT3. Overall, our study highlights the inhibitory role of PIMs in human Th17 cell differentiation, thereby suggesting their association with autoimmune phenotypes.

## Highlights

• IL6/STAT3 axis promotes PIM expression in human Th17 cells
• PIMs inhibit Th17 differentiation
• PIMs suppress Th17 TF *RORA* while promoting Th1 TF *TBX21* within the same cells
• Specific downstream targets of PIMs negatively influence Th17 cell differentiation

## Graphical abstract

**Figure:**
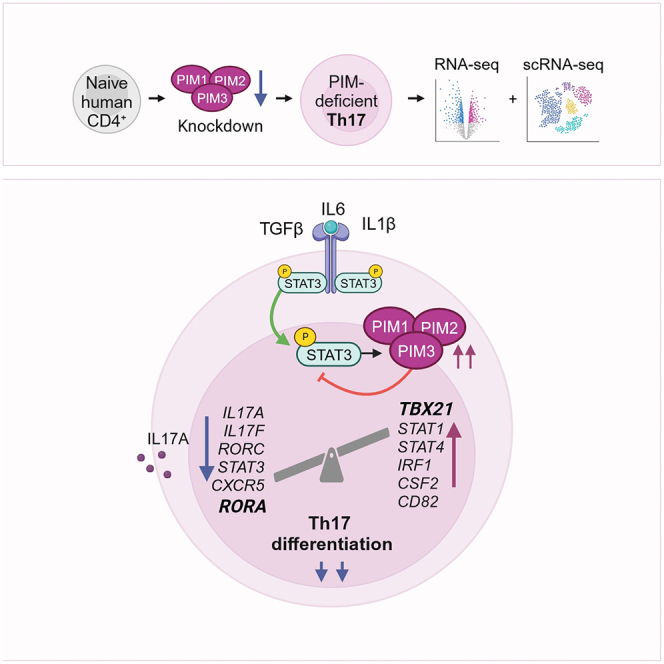

## Teaser

Buchacher et al. show that the three PIM kinases inhibit early human Th17 cell differentiation, by suppressing key Th17 -related genes while promoting the expression of Th1 lineage-specific genes. PIMs control this Th1/Th17 axis, potentially via the STAT family proteins, STAT1 and STAT3, suggesting their involvement in inflammatory phenotypes.

## Introduction

IL17-producing CD4^+^ T helper cells (Th17 cells) play an essential role in mucosal host defense against extracellular pathogens. However, depending on environmental and intrinsic cues, Th17 cells are able to adopt pathogenic states^1^^,^^2^ commonly associated with autoimmune diseases (psoriasis, rheumatoid arthritis [RA], and multiple sclerosis) and cancer.^3^ Th17 cells display distinct gene expression programs when induced *in vitro* by polarizing CD4^+^ T cells with either interleukin (IL)6 and transforming growth factor β (TGFβ) or IL1β/IL23.^4^^,^^5^ Still, the molecular pathways that regulate the switch between pathogenic and nonpathogenic Th17 cells are poorly understood. It’s also unclear how they transiently adopt to other Th cell fates (e.g., Th1 or regulatory T cells [Treg]). A comprehensive investigation of such regulatory pathways that dictate early Th17-fate is crucial for understanding immunopathologies in humans.

The Moloney murine leukemia virus (PIM) family of short-lived oncoproteins includes PIM1, PIM2, and PIM3, which have been extensively studied in hematologic malignancies and solid tumors.^6^ PIMs are conserved and share sequence homology (>60%).^7^ Unlike other kinases, PIMs lack a regulatory domain and are constitutively active.^8^ PIM kinases mediate their oncogenic activity by phosphorylating a wide range of shared substrates that promote cell migration, proliferation, differentiation, and survival^9^ and, thus, are a promising therapeutic target. Small molecule pan-PIM inhibitors that target all three PIMs are in clinical development in oncology.^10^ However, pan-PIM inhibitors used as monotherapy in clinical trials show limited efficacy.^11^^,^^12^ Emerging evidence now indicates that PIM kinases promote immune escape by regulating both tumor and immune cells.^13^^,^^14^ Therefore, combination strategies using pan-PIM inhibitors coupled with clinically approved immunotherapies are being highly discussed. In fact, recent reports on tumor-bearing mice have shown that combining PIM inhibition with adoptive T cell and immune checkpoint therapy causes an increase in central memory T cell phenotype, while promoting long-term tumor control.^15^

Although the effects of PIM inhibition have been exhaustively studied in murine tumors, little is known about the function of these kinases in T cells, especially in human. PIM kinases are regulated primarily by the JAK/STAT pathway, upon T cell receptor (TCR) signaling.^16^^,^^17^ Previously, we showed that the expression of PIMs is enhanced by cytokines inducing human Th1, but not Th2, cell differentiation.^18^ Importantly, we also found PIM kinases to promote early Th1 cell differentiation by upregulating T-box transcription factor TBX21 (TBET*)* and interferon (IFN)γ expression.^19^ Additionally, PIMs are also reported to be downstream of other cytokine signaling pathways known to drive autoimmune diseases (e.g., psoriasis, inflammatory bowel disease, lupus nephritis, and RA).^17^^,^^20^^,^^21^^,^^22^ However, their precise role in human Th17 cell regulation remains unknown.

In the present study, we comprehensively investigated how PIM kinases alter transcriptional gene regulation during early human Th17 cell differentiation. By combining targeted small interfering RNA (siRNA)-mediated PIM triple knockdown with bulk and single-cell RNA sequencing (scRNA-seq), we elucidated the global gene expression changes that are simultaneously induced by the three PIMs. During early Th17 cell differentiation, their expression was driven by the IL6/STAT3 signaling axis. Interestingly, PIM deficiency promoted the expression of genes involved in initiation of Th17 differentiation while suppressing those genes important for Th1 lineage. We further found that PIMs control this Th1/Th17 axis, potentially via the STAT family proteins, STAT1 and STAT3. Our study highlights the inhibitory role of PIMs in human Th17 cell differentiation, suggesting their involvement in inflammatory phenotypes.

## Results

### PIM kinases are upregulated in Th17 cells via the IL6/STAT3 axis

**Figure 1:**
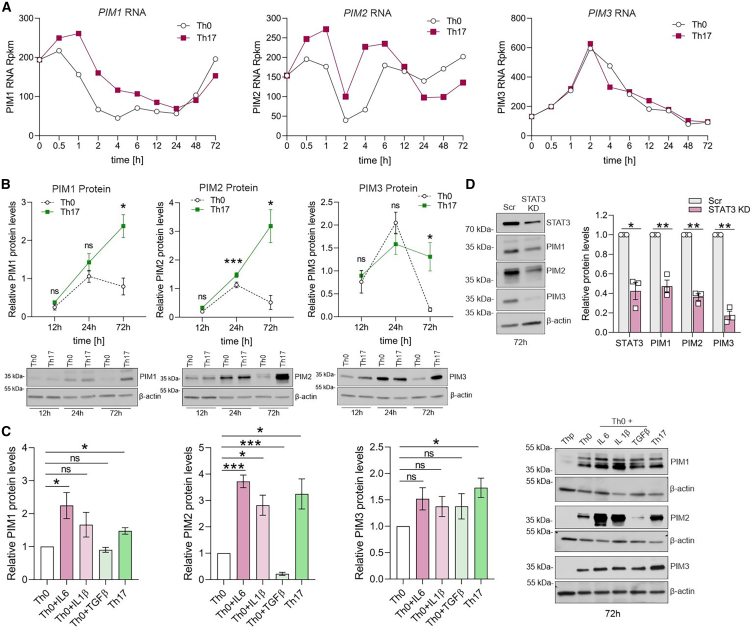
PIMs are upregulated in Th17 cells via the IL6/STAT3 axis (A) Reads per kilobase of transcript, per million mapped reads (Rpkm) of *PIM1*, *PIM2*, and *PIM3* are depicted at different times of activation (Th0) or Th17 differentiation from three biological replicates, using our published RNA-seq data (GEO: GSE52260).^23^ (B) The expression of the three PIMs in Th0 and Th17-polarizing cells over time was analyzed by western blot (bottom). Band intensities of target proteins from four biological replicates were normalized to β-actin (top). (C) Representative western blots of PIM1, PIM2, and PIM3 are shown from naive CD4^+^ T cells cultured for 72 h under activated Th0 condition, Th17 differentiation, or activated Th0 in the presence of Th17 cytokines (IL6, IL1β, and TGFβ) (right). Graphs on the left show band intensities of target proteins from four biological replicates, normalized to β-actin and relative to Th0. Statistical significance was calculated by comparing each condition to Th0. (D) Western blots of STAT3, PIM1, PIM2, and PIM3 protein levels in non-targeting (Scr) vs. STAT3 KD cells, at 72 h of Th17 polarization are shown (left). Protein intensities of STAT3 and PIM kinases from three biological replicates were normalized to β-actin and relative to Scr (right). Graphs in (B)–(D) show mean ± SEM. Statistical significance was calculated using two-tailed Student’s t test (^∗^p < 0.05, ^∗∗^p < 0.01, ^∗∗∗^p < 0.001).

To study the role of PIM kinases in Th17 cells, we first analyzed their RNA expression using RNA-seq data from our previous study.^23^ Here, naive CD4^+^ T cells from human umbilical cord blood were stimulated with anti-CD3/CD28 and Th17-polarizing cytokines (IL6, IL1β, and TGFβ) and analyzed to determine global gene expression in developing human Th17 cells. Cells activated with anti-CD3 and anti-CD28 alone were controls (Th0). Th17 cells had modestly more *PIM1* and *PIM2* transcripts than Th0 cells at early stages of differentiation. *PIM3* transcripts peaked at 2 h for Th0 and Th17 cells (Figure 1A). The PIM expression was further validated at protein level. At 72 h of differentiation, Th17 cells showed significantly more PIM expression than Th0 cells (Figure 1B).

In T cells, PIM expression is regulated downstream of TCR signaling and the cytokine-induced JAK/STAT pathway.^16^^,^^17^ To determine which of the Th17-polarizing cytokines increase PIM expression above TCR-induced levels (Th0), naive CD4^+^ T cells were activated with individual Th17 cytokines and PIM levels were measured at 72 h by western blot (Figure 1C). IL6 induced the expression of PIM1 and PIM2, and slightly PIM3. Given the importance of IL6/STAT3 signaling in establishing Th17 cell identity,^24^^,^^25^ PIM1, PIM2, and PIM3 levels were examined in STAT3-depleted Th17 cells, using western blot analysis. STAT3 deficiency reduced all three PIMs (Figure 1D), as previously reported on PIM1 and PIM2.^26^ Moreover, depletion of the AP-1 transcription factor (TF) basic leucine zipper ATF-like TF (BATF), which is downstream of STAT3 signaling and essential for Th17 cell differentiation,^27^^,^^28^ reduced the expression of the PIMs (Figure S1A). This led us to revisit the BATF chromatin immunoprecipitation sequencing data from our previous study.^27^ We found BATF occupancy in the intronic and intergenic regions of PIM1, PIM2, and PIM3 (Figure S1B), suggesting it regulates PIM transcription. Taken together, the upregulation of PIM kinases in Th17 cells suggests their potential function in modulating Th17 cell differentiation.

### PIM kinases negatively regulate expression of IL17 and RORC

**Figure 2:**
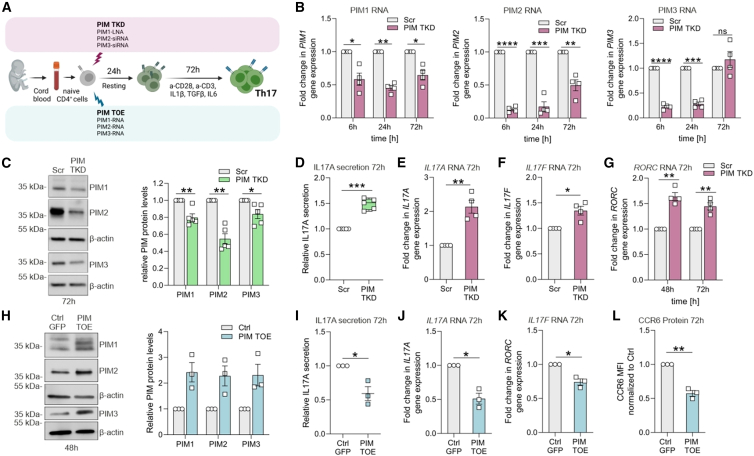
PIMs negatively regulate expression of *IL17* and *RORC* (A) Workflow. Naive CD4^+^ T cells were simultaneously transfected with a pool of one LNA and two siRNAs each targeting PIM1, PIM2, and PIM3, respectively, (TKD), or with *in vitro* transcribed PIM1, PIM2, and PIM3 RNAs (TOE). After 24 h resting, cells were cultured under Th17 conditions for 72 h. (B and C) PIM TKD efficiency was confirmed at RNA level at 6 h, 24 h, and 72 h post-differentiation in four biological replicates using qRT-PCR (B) or at protein level at 72 h of Th17 cell differentiation by western blot (C, left). Band intensities of PIM kinases from five biological replicates were normalized to β-actin and relative to Scr control (C, right). (D) Secreted IL17A cytokine levels in supernatants of PIM TKD Th17 cells are shown at 72 h of polarization. Boxplot represents median and interquartile range, and whiskers extend to maximum and minimum values. Data represent five biological replicates. (E–G) *IL17 A/F* RNA levels at 72 h (E and F) and *RORC* RNA levels at 48 and 72 h (G) in PIM TKD Th17 cells were analyzed in four biological replicates using qRT-PCR. (H–L) PIM overexpression was confirmed at 48 h of polarization by western blot (H, left). Band intensities of PIM kinases from three biological replicates were normalized to β-actin and relative to GFP control (H, right). (I–L) IL17 secretion (I), *IL17A*, and *RORC* RNA expression (J and K) and CCR6 surface expression (L) in PIM TOE Th17 cells at 72 h of polarization, were assessed by ELISA, qRT-PCR, and flow cytometry analyses, respectively, for three biological replicates. (L) Mean fluorescence intensity (MFI) values were normalized to Scr control. ELISA values in plots (D) and (I) were normalized for cell count (live), and then normalized to Scr or GFP control, respectively. (B), (E), (F), (G), (J), and (K) depict transcript FC normalized to control. Plots in (B, C, E–L) show mean ± SEM. Statistical significance is calculated using two-tailed Student’s *t*-test (ns not significant, ^∗^p < 0.05, ^∗∗^p < 0.01, ^∗∗∗^p < 0.001, ^∗∗∗∗^p < 0.0001). See also Figure S2.

To determine the precise role of PIMs in early human Th17 differentiation, we simultaneously silenced the three kinases (triple knockdown [TKD]) and studied its effect on IL17 and the transcriptional regulator retinoic acid-related orphan receptor (*RORC*), which are key markers of the Th17 lineage (Figure 2A). Transient silencing of PIMs reduced RNA expression of *PIM1*, *PIM2*, and *PIM3* at early time points (6 h and 24 h) (Figure 2B). At protein level, 72 h differentiated cells showed significant downregulation of all three PIMs (Figure 2C). Interestingly, PIM silencing increased both *RORC* and *IL17* RNA and intracellular protein expression as well as IL17 cytokine secretion at 72 h of Th17 polarization (Figures 2D–2G and S2A–S2C). Cell survival was not affected, although PIM-deficient Th17 cells showed a slightly greater proliferation rate than controls (Figures S2D and S2E). Further, co-ablation of the PIMs with CRISPR-Cas9 verified the suppressive effect of PIMs on *RORC*, IL17 and CCR6 expression (Figures S2F–S2K). Importantly, findings from these loss-of-function experiments were supported by PIM triple overexpression (TOE), which was carried out using RNAs synthesized by *in vitro* transcription assay (Figures 2A and 2H). *IL17* RNA and cytokine secretion, *RORC* RNA and surface expression of CCR6 were all reduced upon PIM TOE (Figures 2I–2L and S2L). Altogether, these results indicate a negative influence of PIM kinases on human Th17 differentiation.

### PIM kinases alter Th17-specific gene targets

**Figure 3:**
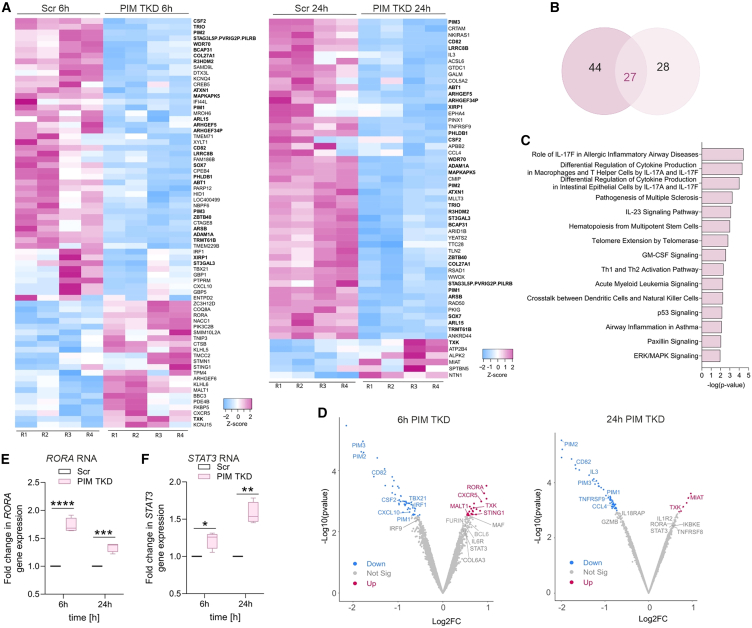
PIMs alters Th17 gene expression (A) *Z* score heatmaps standardized with a FDR of <0.1 and FC of >1.4 for the differentially expressed genes detected at 6 h and 24 h are shown for four biological replicates. Genes common between the two time points are highlighted in bold. (B) Venn diagram demonstrating the number of overlapping differentially expressed genes in PIM TKD Th17 cells at 6 h and 24 h of polarization with a FDR of <0.1 and a FC of >1.4. (C) IPA was used to identify signaling pathways that are significantly altered upon PIM TKD. For analysis, differentially expressed genes at 6 h and 24 h were merged and the top enriched pathways related to T cell signaling and immune-mediated diseases are shown. (D) Volcano plots highlight the Th17-associated transcripts that are differentially expressed upon co-depletion of PIM1, PIM2 and PIM3, at 6 h (left) and 24 h (right) of Th17 polarization with a FDR of <0.1 and a FC of >1.4. Upregulated genes are in pink, and downregulated genes are in blue. Genes colored in gray are selected by a FDR of <0.25. (E and F) *RORA* (E) and *STAT*3 (F) gene expression was analyzed in PIM-depleted Th17 cells at 6 h and 24 h by qRT-PCR. FC normalized to the Scr control was plotted for four biological replicates. Boxplots represent median and interquartile range, and whiskers extend to maximum and minimum values. Statistical significance is calculated using two-tailed Student’s t tests (^∗^p < 0.05, ^∗∗^p < 0.01, ^∗∗∗^p < 0.001, ^∗∗∗∗^p < 0.0001). See also Figure S3 and Table S1.

To assess the combined gene targets of PIM proteins in early-differentiating Th17 cells, we performed bulk RNA-seq on PIM TKD Th17 cells in four biological replicates. Depletion of the PIMs was associated with changes in expression of 71 and 55 genes at 6 h and 24 h of polarization, respectively (false discovery rate [FDR] of <0.1 and fold change [FC] of >1.4). Among these, 27 genes were common at both time points and regulated in the same direction (Figures 3A and 3B and Table S1).

The transcriptome profiling further supported the negative role of PIMs in regulating early human Th17 cell function (Figure 3A). *RORA*, a key Th17 lineage-promoting TF,^29^^,^^30^ was among the top upregulated genes in PIM-depleted Th17 cells (Figures 3A and 3D). PIM silencing increased expression of *STING1*,^31^ *CXCR5*,^24^^,^^32^ *MALT1*,^33^ and *MIAT*,^32^^,^^34^ which are involved in mouse or human Th17 cell induction. Incidentally, PIMs supported the expression of genes that promote a pro-inflammatory Th1 response (*TBX21*, *CSF2*, *IL3*, and *CCL4*), which partially overlaps with genes specifying pathogenic Th17 fate and autoimmune development.^4^^,^^35^ PIMs positively correlated with other Th1 factors like *IRF1*,^36^ *CXCL10*,^37^ and the Th17 repressor gene, *TNFRSF9* (Figures 3A and 3D).^38^

Ingenuity pathway analysis (IPA) showed several genes associated with IL23 signaling, granulocyte macrophage colony stimulating factor signaling, Th1/Th2 activation, ERK/MAPK signaling, and autoimmune processes to be affected by PIM TKD (Figure 3C). Moreover, by lowering the FDR threshold (<0.25), we identified additional Th17-related genes (Figure 3D). Among them, PIMs seemed to repress the TFs *STAT3*^24^^,^^25^ and *MAF*,^39^ the kinase *IKBKE*,^40^^,^^41^ and the surface receptors *IL6R*,^42^ *TNFRSF8*,^43^ and *IL1R2*.^24^ All are critical in priming and/or maintaining Th17 development in human or mouse (Figure 3D). *BCL6*,^44^ *FURIN*,^45^ and *COL6A3*,^32^ which are associated with other Th cell fates, were negatively regulated by PIMs (Figure 3D). PIMs also positively regulated the pro-inflammatory genes *GZMB*^4^ and *IL18RAP*.^46^

Next, we validated the expression changes of selected PIM RNA-seq targets implicated in Th17 cell function. Upregulation of *RORA* was confirmed in PIM TKD Th17 cells by qRT-PCR (Figure 3E). In addition, CXCR5, a STAT3-induced target in Th17 cells,^24^^,^^47^ was upregulated upon PIM depletion at 24 h of differentiation by flow cytometry (Figure S3A). Co-depletion of PIMs increased STAT3 RNA (Figure 3F) and protein levels (Figure S3B) and STAT3 phosphorylation (Y705) (Figure S3C). This inverse association was confirmed by PIM-overexpressed Th17 cells by western blotting (Figure S3D), suggesting a negative feedback loop between PIMs and STAT3 during human Th17 cell differentiation.^48^ We additionally performed qRT-PCR analysis to validate other targets that were associated with Th cell lineage specification (*IL3*, *MIAT*, and *IRF1*) (Figures S3E–S3G). Collectively, these findings indicate that PIM kinases regulate early during human Th17 cell differentiation.

### PIM kinases regulate Th1/Th17 signaling axis during early Th cell differentiation

**Figure 4:**
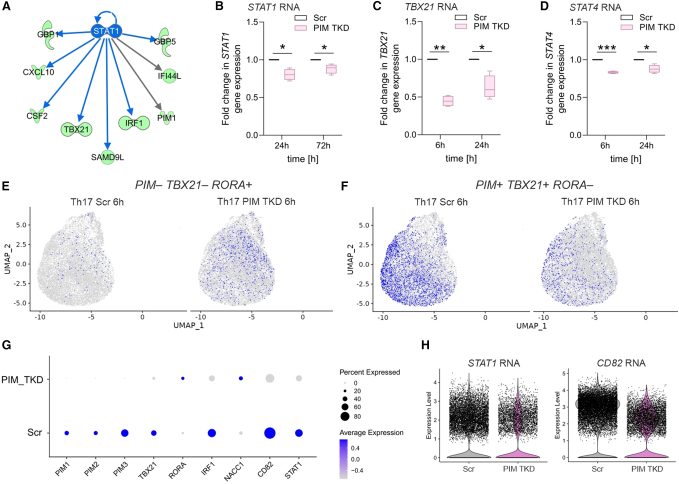
PIMs regulate Th1/Th17 signaling axis during early Th cell differentiation (A) Based on bulk RNA-seq data, STAT1 was predicted as a positive upstream regulator of the differentially expressed genes at 6 h upon PIM TKD using the IPA “upstream regulator” prediction tool (*Z* score < −2, which indicates predicted upstream regulators; gray arrows represent effects not predicted. (B) The downregulation of *STAT1* RNA levels was confirmed at 24 h and 72 h in PIM TKD Th17 cells by qRT-PCR. (C and D) The RNA expression of Th1-related factors *TBX21* (C) and *STAT4* (D) was validated in PIM TKD at 6 h and 24 h of Th17 cell differentiation by qRT-PCR. FC normalized to the Scr control was plotted for four biological replicates. Boxplots represent median and interquartile range, and whiskers extend to maximum and minimum values (B–D). Statistical significance is calculated from four biological replicates using two-tailed Student’s t tests (^∗^p < 0.05, ^∗∗^p < 0.01, ^∗∗∗^p < 0.001). (E and F) scRNA-seq was performed at 6 h in PIM TKD and control Th17 cells using the single-cell fixed RNA profiling. Cells double-negative for *PIM*^*–*^*/TBX21* but *RORA* positive (E) and single cells double-positive for *PIM/TBX21* but *RORA*-negative (F), were colored in blue and projected into a two-dimensional map using UMAP. (G) Differential expression analysis was performed between Scr (control) and *PIM*^*–*^ in PIM TKD samples at 6 h of differentiation (FDR of <0.05, log2FC of >0.24) for one biological replicate. Scaled scRNA-seq dot plot depicting the differentially expressed genes of interest on the x-axis. The color scale represents the average expression of a given gene in the cluster, and the size of the dot represents the percent of cells that express a given gene. (H) Violin plots showing the expression of *CD82* and *STAT1* in Scr and enriched *PIM^–^* cells of PIM TKD samples at 6 h. See also Figures S4 and S5 and Table S2.

We previously reported that *PIM* genes promote Th1 differentiation through STAT4 and other key Th1-specific factors.^19^ IPA upstream regulator analysis of our RNA-seq data predicted *STAT1* to be an upstream regulator of the PIM targets in Th17 cells (Figure 4A). *STAT1* and seven of its downstream targets associated with Th1 cell activation (*TBX21*, *IRF1*, *CSF2*, *CXCL10*, *GBP1*, *GBP5*, and *IFI44L*) were suppressed in PIM-depleted Th17 cells. This was confirmed by qRT-PCR analysis showing *STAT1*, *TBX21*, and *STAT4* to be significantly downregulated in PIM TKD cells at 6 h and 24 h of differentiation (Figures 4B–4D). TBET and STAT4 were further validated at the protein level, together with the Th1-specific cytokine IFN-ɣ (Figures S4A–S4C).

While *TBX21*, known to suppress Th17 function,^49^ was downregulated in PIM-depleted cells, *RORA* showed increased expression at 6 h of differentiation (Figures 3A and 3D). Importantly, RORα, functionally overlaps with RORɣt and serves as an early signature gene for the Th17 lineage.^30^ To investigate whether these changes in Th1/Th17 genes occur in parallel within the same cell, we performed scRNA-seq on 6 h PIM-depleted Th17 cells (Figure S5A). At the single cell level, in 53% of the PIM TKD cells, expression of all the three PIMs was undetectable (*PIM*^*–*^), while Scr (non-targeting) control cells only had 26% of *PIM*^*–*^ cells (Figure S5B). Upon assessing single cells with *PIM* and *TBX21* expression, we noticed a negative correlation of these factors with the Th17 marker *RORA* (Figure 4E). Complementing this trend, *PIM/TBX21* double-negative cells were positive for *RORA* (Figure 4F). To specifically assess the gene expression profile of cells lacking all three PIMs, we performed differential expression analysis on *PIM*^–^ and Scr control cells (Table S2). Importantly, the top differentially expressed genes identified in the scRNA-seq (FDR of <0.05, log2FC of >0.24) and bulk RNA-seq (FDR of <0.1, FC of >1.4) data showed an overlap of 20 genes (Figure S5C). In addition to the *PIMs*, *TBX21*, *IRF1*, and *CD82* were among the common top downregulated genes, whereas *RORA* and *NACC1* were upregulated in both datasets (Figure 4G). Single-cell transcriptome analysis revealed additional Th cell-related genes that were suppressed (*FURIN*, *COL6A3*, *KDSR*, *CCR7*, *TCF7*, and *LEF1*) or promoted (*STAT1*, *IRF8*, *IRF9*, *TNFRSF9*, *FOSL1*, and *TNF*) by PIM kinases (Figures 4G and S5E). In agreement with our bulk RNA-seq results and based on IPA analysis, *STAT1* and several of its downstream targets were dysregulated in PIM-Th17 cells (Figures 4H and S5D). These results support our bulk RNA-seq findings and indicate that PIM kinases regulate the Th1/Th17-related genes within the same cells.

### PIM downstream targets STAT1 and CD82 negatively regulate Th17 cell differentiation

**Figure 5:**
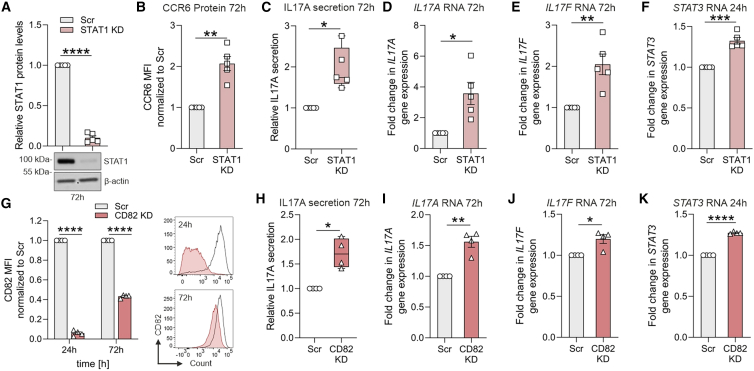
PIM downstream targets STAT1 and CD82 negatively regulate Th17 cell differentiation (A) STAT1 KD efficiency was validated at 72 h in Th17 cells by western blot. Band intensities of STAT1 were normalized to β-actin and relative to Scr control. (B) The surface expression of CCR6 was assessed at 72 h in STAT1 KD Th17 cells. (C) Secreted IL17A cytokine levels in supernatants of STAT1 KD Th17 cells are shown at 72 h of polarization using ELISA. (D–F) *IL17 A/F* RNA levels at 72 h (D and E) and *STAT3* RNA levels at 24 h (F) of differentiation in STAT1-deficient Th17 cells were analyzed by qRT-PCR. Data in (A–F) represent five biological replicates. (G) siRNA-mediated KD of CD82 was validated at 24 h and 72 h in Th17 cells by flow cytometry. Representative histograms are shown (right). (H) Secreted cytokine IL17A levels in supernatants of CD82 KD Th17 cells are shown at 72 h of polarization using ELISA. (I–K) *IL17 A/F* RNA levels (I and J) and *STAT3* RNA levels (K) in CD82 KD Th17 cells, respectively, at 72 h and 24 h, were analyzed using qRT-PCR. (G–K) Data represent four biological replicates. (D–F) and (I–K) depict transcript FC normalized to control. ELISA values in (C) and (H) were normalized for cell count (live), and then normalized to Scr control. Boxplots represent median and interquartile range, and whiskers extend to maximum and minimum values. (B and G, left) Bar plots show the mean fluorescence intensity values normalized to Scr control. Statistical significance is calculated using two-tailed Student’s t tests (^∗^p < 0.05, ^∗∗^p < 0.01, ^∗∗∗^p < 0.001, ^∗∗∗∗^p < 0.0001). Plots in (A), (B), (D–G), (I), and (K) show mean ± SEM. See also Figures S6, S7, and S8.

*STAT1* was found to be one of the targets of PIMs and its expression correlates with PIM RNA levels in Th17 cells (Figures 4A and 4B). Interestingly, gain-of-function mutations in STAT1 inhibit Th17 responses and cause chronic mucocutaneous candidiasis that mimics Th17-mediated immunodeficiencies.^50^^,^^51^^,^^52^ Studies in STAT1-knockout mice confirmed the hyper-Th17 phenotype.^53^^,^^54^ To address the role of STAT1 during early human Th17 cell differentiation, naive CD4^+^ T cells were transfected with STAT1-targeting siRNA and polarized to the Th17 phenotype. Loss of STAT1 significantly increased CCR6, RORɣt, and IL17 expression (72 h) (Figures 5A–5E, and S6A–S6C) and increased the early (24 h) expression of *STAT3* RNA (Figure 5F). These results highlight STAT1-mediated inhibition of Th17 fate.

In addition, to study the additive effect of STAT1 silencing on PIM-deficient Th17 cells, we performed single and double knockdown experiments during the early stages of Th17 differentiation (Figures S6D–S6I). PIM-STAT1 double-deficient Th17 cells showed a modest but significant increase in the expression of *IL17A* mRNA and protein levels compared with Scr controls (Figures S6D–S6F). However, IL17A expression did not change significantly when comparing PIMs/STAT1 single- vs. double-silenced Th17 cells, respectively.

In addition, the expression of PIMs was positively correlated with CD82, the top downregulated gene in our bulk and single-cell RNA-seq data (Figures 3D and 4H). This correlation was reproducibly confirmed in PIM TKD cells using flow cytometry (Figure S7A). CD82, a tumor suppressor gene, mediates connections between cell adhesion, the cytoskeleton, and signaling complexes.^55^ In human T lymphocytes, CD82 acts as a co-stimulatory molecule.^56^^,^^57^ However, its role in Th cell differentiation remains unknown. We found CD82 surface expression to be differentially upregulated by a marginal FC in Th17 cells (compared with Th0) (Figure S7B). Interestingly, CD82-deficient Th17 cells (Figure 5G) showed enhanced mRNA expression of *IL17A*, *IL17F*, and *STAT3* (Figures 5I–5K) and increased RORɣt and IL17 expression, as well as IL17 cytokine secretion (Figures 5H and S7C–S7E). However, no additive effect of CD82 silencing was observed on PIM-deficient Th17 cells (Figures S8A–S8G). The expression of *IL17A*, *STAT3*, and *STAT1* mRNA (Figures S8A, S8D, and S8E), as well as the secretion of IL17A and its intracellular staining (Figures S8B and S8C) were not significantly altered in PIMs-CD82 double-deficient cells, compared with CD82/PIMs single-deficient Th17 cells or Scr control. Overall, the above findings indicate that PIMs may have a role in regulating STAT1 and CD82 expression in human Th17 cells. Moreover, we could confirm that PIM-regulated genes *STAT1* and *CD82*, influence Th17 differentiation in a fashion similar to PIMs.

### Gene expression of PIM kinases and CD82 in healthy and disease datasets

**Figure 6:**
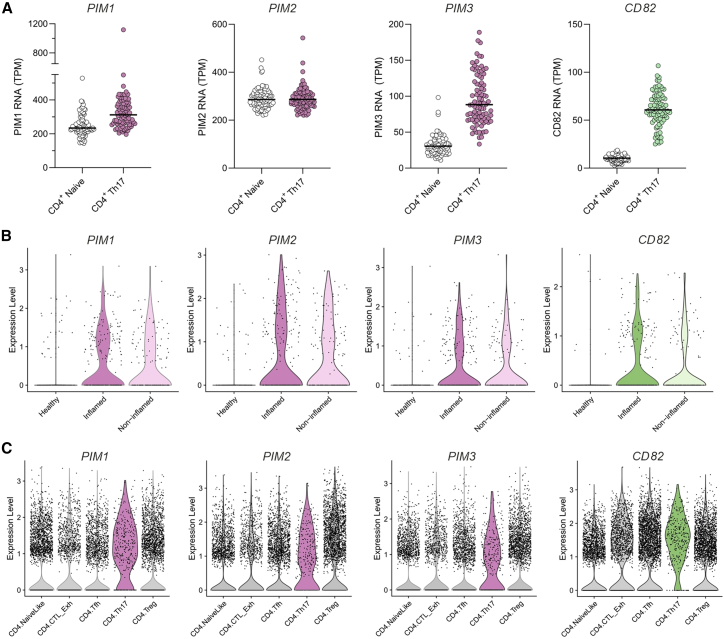
Gene expression of PIMs and CD82 in healthy and disease datasets (A) Gene expression of *PIMs* and *CD82* in peripheral naive CD4^+^ and Th17 memory cells are shown from 91 healthy human subjects using the Database of Immune Cell Expression.^58^ Transcripts per million (TPM) are represented as dot plots for individual subjects with median. (B and C) The expression levels of *PIMs* and *CD82* are shown as violin plots using the scRNA-seq atlas of cycling T cells of 18 patients with UC (inflamed and non-inflamed adjacent tissue) and 12 healthy individuals^59^ (B) and T cells in 21 cancer types from more than 300 patients^60^ (C).

To study the expression of PIMs and CD82 in healthy human peripheral Th17 cells, we used the publicly available Database of Immune Cell Expression (Expression quantitative trait loci and Epigenomics) database.^58^ We found *PIMs* and *CD82* to be upregulated in peripheral Th17 memory cells compared with naive CD4^+^ T cells, indicating their role in human Th17 cells (Figure 6A). Next, to determine the correlation of PIM expression with immune-associated diseases, we explored their transcript profiles in published scRNA-seq datasets from tumor-infiltrating T cells and autoimmunity (ulcerative colitis [UC]). We used the scRNA-seq atlas of human T cells from patients with UC.^59^ *PIMs* and *CD82* were expressed more in cycling T cells of patients with UC than healthy controls (Figure 6B). Also, we investigated these targets in the scRNA-seq atlas of T cells in 21 cancer types from more than 300 patients.^60^ The three *PIMs* with *CD82* were upregulated specifically in Th17 cells when compared with naive, T follicular helper (Tfh), Treg, and exhausted cytotoxic T cells within the CD4^+^ fraction of patients with cancer (Figure 6C). The clinical relevance of increased Th17 cell numbers in tumor progression has been investigated in UC-associated cancer. These Th17 cells, in patients with existing autoimmune conditions, produce cytokines that promote inflammation and cancer progression.^61^ Although it is tempting to speculate the involvement of PIMs, their parallel effects reported on tumor progression makes them a difficult target in disease control.

## Discussion

PIM kinases have received increasing attention as a cancer therapeutic targets owing to their aberrant expression profile across cancer types. However, most of the studies, focusing on their clinical impact, have been obtained from mouse models. Due to the functional redundancy of the three PIM kinases, small molecule inhibitors targeting all three PIM proteins have been developed. To date, limited success of pan-PIM inhibitors in clinical trials has been reported, primarily due to toxicity and off-target phenotypes.^10^ Only a few studies have recently focused on investigating the specific contribution of PIM kinases in immune dysregulation.

Here, we assessed the role of PIMs in gene regulation during early human Th17 cell differentiation. Using PIM triple silencing or overexpression, we found PIMs limit expression of key Th17 marker genes (*IL17A*, *IL17F*, and *RORC*) as well as other Th17-associated factors (*RORA*, *MALT1*, *MIAT*, *CXCR5 TNFRSF9*, *STAT4*, *IL3*, and *STAT3*), thereby confirming that they jointly instruct the initial stages of human Th17 differentiation.

Transcriptome analysis of PIM-depleted Th17 cells found this protein family to affect multiple genes associated with disease etiology and T cell function, including *NACC1*, *KDSR*, *FURIN*, *FOSL1*, and *IRF8*. 3-Ketodihydrosphingosine reductase (KDSR) is known to participate in ceramide synthesis, and mutations in the gene have implications in mendelian disorders of keratinization.^62^^,^^63^ FURIN is a pro-protein convertase that plays a crucial role in adaptive immunity. Its deficiency in T cells affects Treg function, impairs peripheral immune tolerance, and promotes Th2 cell differentiation.^45^^,^^64^ Our former Th17 proteomics analysis showed both KDSR and FURIN to be differentially upregulated in Th17 cells.^65^ Importantly, the present study found these proteins to be repressed by PIM kinases, thereby suggesting that these factors may support the Th17 lineage. FOSL1 and IRF8, which inhibit Th17 cell function, were positively regulated by PIM. We previously reported that FOSL1 acts with its paralog protein FOSL2 for suppressing Th17 function in human.^27^ IRF8 was shown to interact with RORγt to restrain Th17 cell differentiation and colitis in mice.^66^ PIM deficiency further promoted expression of the nucleus accumbens-associated protein 1 (*NACC1*), a TF that is upregulated under pro-inflammatory conditions and promotes autoimmunity by destabilizing FOXP3 expression and suppressing Treg-mediated tolerance.^67^ The precise role of *NACC1* in Th1 or Th17 effector cells warrants further investigation.

Th17 cells are a highly heterogeneous cell population with a spectrum of functional states ranging from conventional to a pathogenic pro-inflammatory phenotype.^68^ Accumulating studies have shown that pathogenic Th17 cells upregulate Th1 marker genes in human and mouse disease models.^4^^,^^69^ While murine studies have provided insights into the transition from a protective to a pathogenic Th17 fate, that switch is poorly characterized in human. We found that PIMs induce the Th1-specific TFs *TBX21* and *STAT4* along with multiple genes (*CSF2*, *IL3*, *CCL4*, *GZMB*, and *TNF*), supporting Th17 pathogenicity.^4^^,^^5^ In addition, PIMs inhibit the expression of *STING1*, which restrains Th17 pathogenicity in mice and induces a protective profile.^70^ Our results also showed that PIM kinases promote the expression of several pro-inflammatory signaling factors including *TNF*,^71^ *CXCL10*,^37^^,^^72^ *CCL20*,^73^ and *CXCL8*,^74^ which influence inflammatory T cells in autoimmune phenotypes, indicating that PIMs are essential in modulating disease responses.

Additionally, we found PIM protein family to affect genes involved in the development of other Th cell fates. For instance, PIMs suppressed Tfh-related genes (*CXCR5*, *BCL6*, *TCF7*, and *LEF1*).^75^ Among these, TCF7 and LEF1, two Wnt signaling targets, were recently found to induce stem-like Th17 cells, with high self-renewal potential.^76^ TCF7 may also serve as a gatekeeper to preserve lineage identity and the stability of effector T cell lineages.^77^ PIM-mediated regulation of these factors complies with murine studies,^15^ thus highlighting the potential of targeting these kinases in sustaining T cell functionality. Interestingly, the recently identified transition of homeostatic, stem-like Th17 cells to pathogenic Th17 cells that drive autoimmune disease,^78^ opens up new avenues for therapeutic intervention.

STAT3 is critical for Th17 cell regulation in human and mouse.^24^^,^^79^^,^^80^ In the current study, the three PIMs were predominantly induced by the IL6/STAT3 signaling axis in Th17 cells while negatively regulating STAT3 expression. This is in line with previous studies showing PIM1 to be downstream of STAT3 signaling,^16^^,^^26^ where it forms a negative feedback loop with the JAK/STAT pathway for tight regulation of its own expression and function.^48^ We additionally found PIMs to inhibit Th17-related genes that are activated by STAT3 (*RORA*, *CXCR5*, *IL1R2*, *MALT1*, and *COL6A3*).^24^ Upregulation of STAT3 in PIM-deficient Th17 cells further negatively correlated with STAT1 expression and other Th1-associated genes (*STAT4*, *CSF2*, and *TBX21*). STAT3 was shown to competitively reduce STAT1 activation following Th17 cell-promoting cytokine stimulation.^81^ Deletion of STAT3 in murine T cells enhanced STAT1 activity and gene expression. These opposing actions of STAT1 and STAT3 were demonstrated in IFN-γ expressing CD4^+^ T cells *in vivo* during chronic lymphocytic choriomeningitis infection.^82^ Thus, negative regulation of STAT1 activation may be an important function of STAT3 in maintaining Th17 cell populations.

Emerging evidence highlights the involvement of PIM kinases in the regulation of inflammatory diseases. Recently, Maney et al. found PIM1 expression to be significantly higher in synovial CD4^+^ T cells of patients with early RA than controls. Still, pan-PIM inhibition of CD4^+^ T cells isolated from these patients showed little change in IL17 production in response to re-stimulation.^83^ Use of pan-PIM inhibitors, however, requires careful assessment owing to well-reported off-target phenotypes.^84^^,^^85^^,^^86^ Inhibitors at higher doses can cause clinical complications.^87^ Surprisingly, reports have found potential oncogenic inhibitors to kill cancer cells even when the target protein is knocked out. This indicates the need for better scrutiny when verifying cellular targets of inhibitors in clinical development.^88^^,^^89^ The current study thus profiles specific changes in the transcriptome of Th17 cells using targeted perturbation of PIMs.

### Limitations of the study

Here, PIMs were investigated in conventional human Th17 cells at the early stages of differentiation. However, the current study limits its investigation to healthy individuals only. It is equally important to shed light on the role of PIMs in Th17 maintenance and terminal differentiation, especially in the context of disease pathogenesis. Since PIMs restrain inflammatory cytokine secretion in Th17 cells, exploring their function in Treg-mediated immune suppression is imperative. In addition, PIMs act at the interface of cancer and immune cells, and thus, it would be important to investigate their participation in tumor infiltrating T cell responses. Furthermore, the current study lacks substrate analysis to identify PIM-specific targets during the early stages of human Th17 differentiation. Such analysis could help to better understand how PIM kinases regulate the expression of several TFs. Substrate analyses of both Th1 and Th17 cells could reveal potential differences in PIM targets between the two Th subsets. However, to specifically label PIM substrates, a chemical genetic screen coupled with a peptide capture mass spectrometry-based approach is needed, which could be challenging to perform in primary human T cells.

## STAR★Methods

### Key resources table

**Table:** 

REAGENT or RESOURCE	SOURCE	IDENTIFIER
**Antibodies**		

rabbit polyclonal anti-human PIM1	Cell Signaling Tech	Cat# 2907; RRID: AB_2283785
rabbit monoclonal anti-human PIM2	Cell Signaling Tech	Cat# 4730; RRID: AB_2163921
rabbit monoclonal anti-human PIM3	Cell Signaling Tech	Cat# 4165; RRID: AB_1904094
rabbit monoclonal anti-human BATF	Cell Signaling Tech	Cat# 8638; RRID: AB_11141425
rabbit monoclonal anti-human STAT1	Cell Signaling Tech	Cat# 9172; RRID: AB_2198300
rabbit monoclonal anti-human STAT3	Cell Signaling Tech	Cat# 9139; RRID: AB_331757
rabbit monoclonal anti-human STAT4	Cell Signaling Tech	Cat# 2653; RRID: AB_2255156
mouse polyclonal anti-human TBET	Santa Cruz Biotechnology	Cat# sc-21749; RRID: AB_628331
mouse monoclonal anti-human β-Actin	Sigma-Aldrich	Cat# A5441; RRID: AB_476744
HRP goat anti-rabbit IgG	BD Biosciences	Cat# 554021; RRID: AB_395213
mouse IgGκ BP-HRP	Santa Cruz Biotechnology	Cat# sc-516102; RRID: AB_2687626
anti-human CD45RO PE	BD Bioscience	Cat# 555493; RRID: AB_395884
anti-human CD45RA FITC	BD Bioscience	Cat# 555488; RRID: AB_395879
anti-human CD82 Alexa Fluor 647	BD Bioscience	Cat# 564341; RRID: AB_2738755
anti-human CXCR5 Alexa Fluor 488	BD Bioscience	Cat# 558112; RRID: AB_397034
anti-human STAT3 PE	BD Bioscience	Cat# 560391; RRID: AB_1645535
anti-human phospho-STAT3 Alexa Fluor 647	BD Bioscience	Cat# 557815, RRID: AB_647144
anti-human IL17A BV421	BD Bioscience	Cat# 562933; RRID: AB_2737902
anti-human RORγt Alexa Fluor 647	BD Bioscience	Cat# 563620; RRID: AB_2738324
PE mouse IgG1, κ isotype	BD Bioscience	Cat# 559320; RRID: AB_397218
Alexa Fluor 488 rat IgG2b, κ isotype	BD Bioscience	Cat# 557726; RRID: AB_396834
BV421 mouse IgG1, k isotype	BD Bioscience	Cat# 562438; RRID: AB_11207319
Alexa Fluor 647 mouse IgG1, κ isotype	BD Bioscience	Cat# 557714; RRID: AB_396823
Alexa Fluor 647 mouse IgG2a, κ isotype	BD Bioscience	Cat# 558053; RRID: AB_1645617
Alexa Fluor 647 mouse IgG2b, κ isotype	BD Bioscience	Cat# 557903; RRID: AB_396928
anti-human CD3	Beckman Coulter	Cat# IM1304; RRID: AB_131612
anti-human CD28	Beckman Coulter	Cat# IM1376; RRID: AB_131624
anti-human IFNɣ	R&D Systems	Cat# MAB285; RRID: AB_2123306
anti-human IL4	R&D Systems	Cat# MAB204; RRID: AB_2126745

**Chemicals, Peptides, and Recombinant Proteins**		

Ficoll-Paque PLUS	GE Healthcare	Cat# 17144003
X-vivo 20 serum-free medium	Lonza	Cat# 04-448Q
Opti-MEM	Gibco	Cat# 31985-062
RPMI 1640 medium	Sigma-Aldrich	Cat# R5886
L-glutamine	Sigma-Aldrich	Cat# G7513
Penicillin-Streptomycin	Sigma-Aldrich	Cat# P0781
Fetal Bovine Serum	Serana	Cat# S-FBS-AU-015
recombinant human IL6	R&D Systems	Cat# 7270-IL
recombinant human IL1β	R&D Systems	Cat# 201-LB
recombinant human TGFβ	R&D Systems	Cat# 240-B
recombinant *S. pyogenes* Cas9-nuclear localization sequence (NLS) purified protein	QB3 MacroLab, University of California, Berkeley	N/A
*XmaI*	Thermo Scientific	Cat# ER0171
*PmeI*	Thermo Scientific	Cat# ER1342
*SpeI*	Thermo Scientific	Cat# ER1252
RIPA buffer	Thermo Fisher	Cat# 89901
Protease and Phosphatase Inhibitor Cocktail	Thermo Fisher	Cat# 1861281
Ionomycin	Sigma-Aldrich	Cat# I0634
PMA	Calbiochem	Cat# 524400
Brefeldin A	Enzo Life Sciences	Cat# BML-G405-0005

**Critical Commercial Assays**		

Dynal CD4 Positive Isolation Kit	Invitrogen	Cat# 11331D
human IL17A Duoset ELISA kit	R&D Systems	Cat# DY317-05; DY008
human IFN-gamma DuoSet ELISA kit	R&D Systems	Cat# DY285B-05; DY008
DC™ Protein Assay Kit II	BioRad	Cat# 5000112
Dead cell removal kit	Milteny	Cat# 130-090-101
Gene Expression Flex assay	10 X Genomics	Cat# CG000527-RevC
T7 mScript™ Standard mRNA Production System	Cell Script	Cat# C-MSC100625
RNeasy Mini Kit	Qiagen	Cat# 74106
Rneasy MiniElute Cleanup Kit	Qiagen	Cat# 74204
Illumina Stranded mRNA Prep	lllumina	Cat# 1000000124518
SuperScript™ II Reverse Transcriptase	Invitrogen	Cat# 18064022
*RORA* Taqman Gene Expression Assay	Applied Biosystems	Cat# Hs00536545_m1
Absolute QPCR Mix, ROX	Thermo Scientific	Cat# AB1139A
FITC Annexin V Apoptosis Detection Kit I	BD Bioscience	Cat# 556547
CellTrace™ Violet Cell Proliferation Kit	Thermo Fisher	Cat# C34557
BD Phosflow Fix buffer I	BD Bioscience	Cat# 557870
BD Phosflow Perm buffer III	BD Bioscience	Cat# 558050
eBioscience™ Foxp3/Transcription Factor Staining Buffer Set	Thermo Scientific	Cat# 00-5523-00

**Deposited Data**		

RNA-seq	This paper	GEO: GSE231650
scRNA-seq	This paper	GEO: GSE231651
RNA-seq data of Th17 cells	Tuomela et al. ^23^	GEO: GSE52260
BATF CHIP-seq data of Th17 cells	Shetty et al. ^27^	GEO: GSE174810
RNA-seq PBMC	Schmiedel et al. ^58^	https://dice-database.org/
Immune scRNA-seq data of patients with ulcerative colitis	Smillie et al.^59^	Single Cell Portal: SCP259
scRNA-seq data of the TILs	Zheng et al.^60^	Figshare: https://doi.org/10.6084/m9.figshare.21981536.v1

**Oligonucleotides**		

Alt-R CRISPR-Cas9 tracrRNA	IDT	Cat# 1072533
siGENOME human CD82 siRNA	Dharmacon	Cat# D-003901-01
siGENOME human STAT1-SMARTpool siRNAs	Dharmacon	Cat# MQ-003543-01
qPCR primer sequences for various genes	This paper	Table S3
siRNA and LNA sequences	This paper	Table S3

**Recombinant DNA**		

plasmid GEM-GFP64A	Zhao et al. ^90^	N/A
plasmid CMV6-PIM1	Origene	Cat# RC205853
plasmid CMV6-PIM2	Origene	Cat# RC201933
plasmid CMV6-PIM3	Origene	Cat# RC212690

**Software and Algorithms**		

GraphPad Prism 8	GraphPad	N/A
Flow Jo	FlowJO LLC	https://www.flowjo.com/
Ingenuity Pathway Analysis	Qiagen	N/A
ImageJ Fiji	NIH	http://imagej.net/software/fiji/
UCSC human reference genome GRCh38	Schneider et al. ^91^	http://genome.ucsc.edu/cgi-bin/hgGateway
FastQC v.0.11.8	Babraham Bioinformatics	https://www.bioinformatics.babraham.ac.uk/projects/fastqc/
TMM normalization, Bioconductor package edgeR 3.32.1	Robinson et al. ^92^ McCarthy et al. ^93^	https://bioconductor.org/packages/release/bioc/html/edgeR.html
Cell Ranger Software v7.0.1	10x Genomics	https://www.10xgenomics.com/support/software/cell-ranger/
Seurat v 4.3.0	Butler et al. ^94^	http://www.satijalab.org/seurat
ILoReg v1.8.0	Smolander et al. ^95^	https://www.bioconductor.org/packages/release/bioc/html/ILoReg.html
Reproducibility Optimized Test Statistic, ROTS Bioconductor package 1.10.1	Suomi et al.^96^	https://bioconductor.org/packages/release/bioc/html/ROTS.html

### Resource availability

#### Lead contact

Further information and requests for resources and code should be directed to the lead contact, Riitta Lahesmaa (rilahes@utu.fi).

#### Materials availability

The study did not generate any materials.

#### Data and code availability

• All data reported in this paper will be shared by the lead contact upon request.
• Raw bulk RNA-seq and scRNA-seq data reported in this paper are available on NCBI Gene Expression Omnibus (GEO) with accession numbers GSE231650 and GSE231651, respectively.
• Any additional information required to reanalyze the data reported in this work paper is available from the lead contact upon request.

### Experimental model and subject participant details

#### Human CD4^+^ T cell isolation and differentiation

Studies with primary human CD4^+^ T cells were approved by the Finnish Ethics Committee. Oral informed consent was obtained from all donors prior the onset of the study. Primary human CD4^+^ T cell isolation and Th17 differentiation were performed as described earlier.^27^ In brief, primary human CD4^+^ T cells were isolated from the umbilical cord blood of healthy neonates (Turku University Central Hospital, Turku, Finland) using the Ficoll-Paque density gradient centrifugation (Ficoll-Paque PLUS; GE Healthcare). CD4^+^ T cells were further enriched using CD4^+^ Dynal positive selection beads (Dynal CD4 Positive Isolation Kit; Invitrogen). Prior to activation, naive CD4^+^ T cells from different donors which were highly positive for CD45RA and negative for CD45RO, characterized by flow cytometry (see Flow Cytometry) were pooled. Cells were activated with plate-bound anti-CD3 (3.75 μg/mL; Beckman Coulter) and soluble anti-CD28 (1 μg/mL; Beckman Coulter) in X-vivo 20 serum-free medium (Lonza) supplemented with L-glutamine (2 mM, Sigma-Aldrich) and antibiotics (50 U/mL penicillin plus 50 μg/mL streptomycin; Sigma-Aldrich). Th17 cells were cultured in the presence of IL6 (20 ng/mL; Roche), IL1β (10 ng/mL; R&D Systems) and TGFβ (10 ng/mL; R&D Systems), in the presence of neutralizing anti-IFNγ (1 μg/mL; R&D Systems) and anti-IL4 (1 μg/mL; R&D Systems) to block Th1 and Th2 differentiation, respectively. As control, cells were activated from naive CD4^+^ T-cells with anti-CD3 and anti-CD28 in the presence of neutralizing antibodies, but without cytokines (Th0).

To study PIM induction by Th17 relevant cytokines, naive CD4^+^ T cells were cultured under the following conditions: CD3/CD28 activation (Th0), Th0 with IL6, Th0 with IL1β, Th0 with TGFβ, and Th17 differentiation conditions for 72 h. The neutralizing antibodies anti-IFNγ and anti-IL4 were added to each of these conditions. All cytokine and antibody concentrations were as described for Th17 culture conditions. PIM1, PIM2 and PIM3 protein levels were estimated by Western blot at 72 h.

#### Gene silencing

Four million CD4^+^ T cells were resuspended in Opti-MEM and transfected with the corresponding gene-targeting small interfering RNA (siRNA) and locked nucleic acid (LNA)-modified antisense oligonucleotide, (the latter was used only when targeting PIM1), using Nucelofector 2C system (Lonza). Control cells were treated with non-targeting or scramble (Scr) siRNA/LNA (Sigma). For single gene knockdown (KD), cells were transfected with 6 μg of target siRNA or 6 μg of Scr siRNA (Table S3). For PIM triple KD (PIM TKD), cells were transfected with a pool of 6.5 μg of PIM-targeting LNA/siRNAs (1.5 μg of PIM1 LNA, 2.5 μg of PIM2 siRNA and 2.5 μg of PIM3 siRNA) or 6.5 μg of non-targeting LNA/siRNA (1.5 μg LNA and 5 μg of siRNA). For the double KD experiments, cells were nucleofected with 12.5 μg of PIMs-STAT1 or PIM-CD82-targeting siRNA-LNA (i.e., 1.5 μg PIM1 LNA, 2.5 μg PIM2, 2.5 μg PIM3 and 6 μg STAT1/CD82 siRNA). 12.5 μg of Scramble siRNA-LNA was used as non-targeting control. Single KD controls for PIM/STAT1/CD82 were also maintained (i.e., 6.5 μg PIMs or 6 μg STAT1/CD82 siRNA and 6 μg or 6.5 μg scramble control siRNA-LNA). After cell resting for 24 h in RPMI 1640 medium (Sigma-Aldrich) supplemented with pen/strep, L-glutamine and 10% FCS, cells were activated and differentiated for 72 h under Th17 conditions. For bulk RNA-sequencing, Scr and PIM TKD Th17 cells were harvested at 6 and 24 h of differentiation.

#### CRISPR-Cas9-mediated PIM ablation

Guide RNAs (gRNAs) were *in vitro*-assembled with the Cas9 protein as described.^97^ Briefly, crisprRNA (crRNA)s, designed using Synthego gRNA design tool and synthesized by IDT, and tracrRNA (Alt-R CRISPR-Cas9 tracrRNA, IDT) were reconstituted to 160 M and combined in equimolar amounts (1:1), followed by incubation at 37°C for 30 min to prepare 80 μM gRNA reagent. Assembled gRNA was then mixed with equal volume of 40 μM recombinant *S. pyogenes* Cas9-nuclear localization sequence (NLS) purified protein (QB3 MacroLab, University of California, Berkeley) (giving 2:1 gRNA to Cas9 molar ratio) together with 1 μL of 100 μM non-homologous single-strand DNA enhancer (ssODNenh), synthesized by IDT, and incubated for 10 min at 37°C for a final concentration of 20 μM CRISPR-Cas9 ribonucleoprotein (RNP). For PIM ablation, a pool of PIM-targeting gRNAs (prepared using the following PIM targeting crRNAs: PIM1_5′-TCTTCGACTTCATCACGGAA-3′, PIM1_5′-CGGGCCTCTCGAACCAGTCC-3′, PIM1_5′-TTCAGCAGGACCACTTCCAT-3′, PIM2_5′-GAGGGGGCCGAGTCGATACT-3′ and PIM3_5′-GGACAAGGAGAGCTTCGAGA-3′) was used. For control cells, non-targeting gRNA was prepared by using negative control crRNA (NC1 from IDT: 5′-CGTTAATCGCGTATAATACG-3′) with tracrRNA. Freshly purified CD4^+^ cells (4 × 10^6^ cells) were then transfected by nucleofection with the RNP complexes and rested for 24 h in RPMI supplemented with 10% serum and further cultured under Th17 conditions, as described above.

#### *In vitro* transcription (IVT) for PIM overexpression

The T7 promoter containing plasmids pGEM-GFP64A^90^ (gift from Prof. R. Morgan, National Institutes of Health, National Cancer Institute, USA), pCMV6-PIM1, pCMV6-PIM2 and pCMV6-PIM3 (all from Origene) were first digested with restriction enzymes to generate linearized templates for IVT. pGEM-GFP plasmid was digested with SpeI, PIM1/PIM3 plasmids with XmaI and PIM2 plasmid with PmeI (all from Thermo Scientific). Next, IVT RNA was produced from these templates using T7 mScript Standard mRNA Production System (Cell Script), by following manufacturer’s instructions. The IVT RNA was purified using RNeasy MiniElute Cleanup Kit (Qiagen).

For the triple over-expression (TOE) experiments, four million cells were transfected with a total of 90 pmol of PIM IVT RNA (30 pmol for each of the three PIMs) or 90 pmol of control GFP RNA. Cells were rested for 18 h post-nucleofection and further cultured under Th17 conditions, as described above.

### Method details

#### Gene expression analysis

##### RNA isolation and bulk RNA-sequencing

RNA-sequencing was performed on four biological replicates of PIM TKD Th17 cells and Scr Th17 cells collected at 6 and 24 h after initiation of Th17 differentiation. RNA was purified using RNeasy Mini Kit (Qiagen), and treated in-column with DNase, according to the manufacturer’s instructions. The quality of the total RNA was ensured with Advanced Analytical Fragment Analyzer (Advanced Analytical Technologies, Heidelberg, Germany). Libraries were prepared, according to Illumina Stranded mRNA Preparation protocol (1000000124518). The sequencing was carried out on Illumina NovaSeq600 sequencing system at the Finnish Functional Genomics Center.

##### Transcriptomics data processing

The quality of the raw sequencing reads was checked with FastQC tool version 0.11.8.^98^ Further analysis was carried using R version 4.1.0 and Bioconductor version 3.13.^99^^,^^100^ The reads were aligned to the UCSC hg38 human genome ref. ^91^ derived from Illumina iGenomes using Rsubread package (version 2.6.4) and its inbuilt Refseq gene annotation.^101^ Rsubread was also used for calculating the genewise read counts. Normalization was performed using TMM (trimmed mean of M-values) method of the edgeR package version 3.34.1.^92^^,^^93^ Paired statistical testing between sample groups was carried out using ROTS version 1.20.0,^96^ and the differentially expressed genes were selected requiring false-discovery rate (FDR) below 0.1 and absolute fold-change (FC) above 1.4 (Table S1).

#### Single-cell RNA-sequencing and data analysis

Naive CD4^+^ cells of three different donors were pooled and PIM TKD and Scr cells were differentiated for 6 h under Th17 condition. Dead cells were removed using the dead cell removal kit (Milteny, #130-090-101) and single-cell suspensions with >85% viability were used for scRNA-seq experiments. scRNA-seq libraries were prepared using Gene Expression Flex protocol from 10 X Genomics, according to User Guide CG000527-RevC. A total number of 300 000 cells per sample were used for the hybridization and 2 individually barcoded samples were pooled together after the hybridization. A total of 8 cycles of PCR was used for library amplification. Sequencing was carried out using 1% of PhiX spike-in and 28 bp, 10 bp, 10 bp and 90 bp read lengths for Read1, i7 index, i5 index and Read 2 respectively on the SP flow cell of the Novaseq6000 instrument (Illumina). Cell Ranger version 7.1 was used to generate FASTQ files.

The data was pre-processed using Cell Ranger (v7.0.1)^102^ and the GRCh38 reference genome (Cell Ranger version ID: 2020-A). Quality control and filtering of high-quality cells were performed using Seurat (v 4.3.0).^94^ Cells with an abnormally high proportion of reads mapped to the mitochondrial genes (more than 10%) and less than 200 expressed genes were filtered out. The data was analyzed as one set of samples. The dataset was further normalized using Seurat’s LogNormalize method. ILoReg (v1.8.0)^95^ and Seurat were used to visualize cell heterogeneity. Seurat was used for scaling and Principal Component Analysis, and Uniform Manifold Approximation and Projection for Dimension Reduction (UMAP)^103^ to visualize the data. Gene expression was visualized with the FeaturePlot, VlnPlot and DotPlot functions of the Seurat package.

Differential expression analysis was performed between PIM TKD Th17 cells which did not express any of the three PIM genes (*PIM*^*−*^), and all cells from the Scr Th17 cells. The Scr sample was downsampled 100 times to obtain the same number of cells for the PIM TKD and Scr samples. For each Scr subset, the DE analysis between the PIM TKD and Scr was performed using the Wilcoxon rank-sum test and correcting the p values for multiple comparisons using the Benjamini-Hochberg method. A column (signif_prop) was added to the result table (Table S2) that denotes the proportion of subsets with FDR < 0.05 and log2FC > 0.24.

#### Data visualization

Heatmaps were plotted using heatmapper.^104^ Targets with FDR < 0.1 and FC > 1.4 are shown and the genes common between the 6 and 24 h time points are marked in bold. Volcano plots were generated using the ‘Volcano Plot’ function on Galaxy Europe.^105^ Th17-related genes are shown with a cutoff FDR < 0.1 and FC > 1.4. Th17 relevant genes with a cutoff FDR above 0.1 but below 0.25 are shown in gray.

#### Functional data analysis

Functional enrichment and upstream regulator analyses were performed using Ingenuity Pathway Analysis (Qiagen). IPA pathways with p value < 0.0.5 were considered to be significantly enriched. The activation *Z* score was calculated to predict activation or inhibition of transcriptional regulators. Upstream regulators with p values < 0.05 and |*Z* score| > 2 were considered to be significantly activated or inhibited.

#### Quantitative real-time PCR

Total RNA was purified as described in the method section *RNA isolation and sequencing*. Single-stranded cDNA was synthesized with the SuperScript II Reverse Transcriptase, according to the manufacturer’s instruction (Invitrogen). TaqMan primers and probes were designed using the Universal Probe Library Assay Design Center (Roche). Quantification of *RORA* mRNA was performed using the TaqMan Gene Expression Assay (Applied Biosystems). EF1α gene was used as endogenous control. Primer and probe sequences are summarized in Table S3. TaqMan reactions were performed using Absolute QPCR Mix, ROX (Thermo Scientific) and followed by qPCR runs on QuantStudio 12K Flex Real-Time PCR instrument and data was analyzed with QuantStudio 12K Flex Real-Time PCR System v1.2.3 software (Thermo Fisher Scientific). Relative quantification of gene expression values was calculated using the ddCt method.

#### Western blots

Cell samples were lysed in RIPA buffer (Thermo Fisher) supplemented with 1X Protease and Phosphatase Inhibitor Cocktail (Thermo). Cell lysates were sonicated on a Bioruptor (Diagenode) and centrifuged at 18,000 × g for 10 min. Protein concentration was determined using DC Protein assay (BioRad). After boiling in 6X loading dye (330 mM Tris-HCl, pH 6.8; 330 mM SDS; 6% β-ME; 170 mM bromophenol blue; 30% glycerol), 20–30 μg of protein were separated on 4–20% Mini-PROTEAN TGX gels (BioRad Laboratories) and transferred to PVDF membranes (*Trans*-blot Turbo Transfer Packs, BioRad Laboratories). Membranes were blocked with 5% BSA-TBST (Tris-buffered saline and 0.1% Tween 20) and incubated with primary antibody overnight at 4°C. After washing with TBST, membranes were incubated with alkaline phosphatase–coupled secondary antibodies diluted (1:5000) in 5% BSA-TBST at RT for 1h. Membranes were cut into sections and probed with antibodies, or the membranes were stripped with stripping buffer (25 mM glycine and 1% SDS, pH 2.5), blocked with 5% BSA-TBST and re-probed with antibodies. The band intensity of each target was quantified using ImageJ (NIH) and normalized to loading control band intensity in each lane. Antibodies are listed in the Key resource table.

#### Flow cytometry

Surface staining of CD82 (Tspan-27) and CXCR5 was performed at 24 and/or 72 h of Th17 differentiation. Cells were washed twice in FACS buffer (2% FBS/0.1% Na-azide/PBS) and incubated with anti-CD82 (Alexa Fluor 647, BD Bioscience) and anti-CXCR5 (Alexa Fluor 488, BD Bioscience) for 30 min at 4°C in dark. CD4^+^ T cells were labeled with fluorescently-conjugated anti-CD45RO (PE, BD Bioscience) and anti-CD45RA (FITC, BD Bioscience). Intracellular staining of total STAT3 and STAT3 phosphorylation was performed on differentiated Th17 cells at 24 and 72 h. Cells were fixed using BD Phosflow Fix buffer I (BD Biosciences) at 37°C for 10 min and permeabilized with BD Phosflow Perm buffer III (BD Biosciences) for 30 min on ice. After washing twice with FACS buffer, cells were stained with fluorescently-conjugated anti-phospho-STAT3 (pY705) (Alexa Flour 647, BD Biosciences) and anti-STAT3 (PE, BD Bioscience), or corresponding isotype control (BD Bioscience). For intracellular IL17A staining at 72 h of differentiation, cells were incubated in X-vivo 20 medium with 50 ng/mL phorbol 12-myristate 13-acetate (PMA) and 500 ng/mL ionomycin (Sigma-Aldrich) and 10 μg/mL Brefeldin A (BD Biosciences) for 5 h. For intracellular detection of IL17A and RORɣt, cells were fixed and permeabilized in Foxp3 fixation buffer kit (Invitrogen), according to the manufacturer’s instructions. Cell viability was determined using the FITC Annexin V Apoptosis Detection Kit I (BD Bioscience). Antibodies are listed in Key resources table. Data from all samples were acquired on BD LSRFortessa (BD Biosciences) and analyzed with Flowjo (FlowJo LLC).

#### Proliferation assay

T cell proliferation was assessed in Scr control and PIM TKD Th17 cells. Post-transfection, naive CD4^+^ cells (Scr and PIM TKD) were labeled with cell trace violet (CTV; Thermo Fisher) according to the manufacturer’s instructions and cultured for 96 h under Th17 polarizing conditions. The percent of CTV positive cells was acquired on BD LSRFortessa (BD Biosciences) and analyzed with Flowjo (FlowJo LLC).

#### Cytokine secretion by ELISA

Secreted IL17A or IFNɣ levels were determined from Th17 cell-culture supernatants at 72 h using the human IL17A Duoset ELISA kit or human IFN-gamma DuoSet ELISA kit (both from R&D Systems). Levels of cytokine secretion was normalized to the number of living Th17 cells determined by forward and side scattering in flow cytometry analysis (BD LSRFortessa flow cytometer; BD Biosciences).

### Quantification and statistical analyses

Figures and statistical analyses were performed with GraphPad Prism8 software (GraphPad Software, Inc.). At least three independent biological replicates were performed for each experiment unless otherwise stated in the figure legend. The number of biological replicates and the related statistical methods are described within figure legends. Statistical significance was calculated using Student’s two-tailed t test for two groups or one-way ANOVA, Tukey’s multiple comparisons test for multiple groups. Statistical significance was concluded when a probability value (p value) was lower than 0.05. The data represent means ± the standard error of the mean (SEM), as indicated in the figures. Graphs are plotted with depicted individual values as dots. Statistical significance was represented as: ^∗^p < 0.05; ^∗∗^p < 0.01; ^∗∗∗^p < 0.001; ^∗∗∗∗^p < 0.0001.

### Additional resources

The transcriptome data of early human Th17 cell differentiation was downloaded from NCBI GEO: GSE52260,^23^ and normalized expression values (RPKM) were extracted and plotted for *PIM1*, *PIM2* and *PIM3*. Bigwig files of BATF CHIP-seq data were downloaded from GEO: GSE174810^27^ and BATF binding over intergenic regions of PIMs were visualized using IGV. The bulk RNA-seq data of naive CD4^+^ and memory Th17 cells from the DICE project was downloaded from https://dice-database.org/.^58^ Normalized expression values (TMP) were extracted and plotted for *PIM*s and *CD82*. The immune scRNA-seq data (genes, barcodes, matrix) and metadata from the ulcerative colitis study were downloaded from the Single Cell Portal: SCP259.^59^ The scRNA-seq data of the TILs was downloaded from figshare: https://doi.org/10.6084/m9.figshare.21981536.v1.^60^ The normalized expression of *PIM*s and *CD82* obtained with the NormalizeData function was plotted with the function VlnPlot using Seuruat V4.3.0 for the cell types of interest (cycling T cells for the UC study and naive, CTL, Tfh, Th17 and Tregs for the CD4^+^ TILs study).

## Supplemental information

Document S1. Figures S1–S8

Table S1. DE genes of PIM TKD, bulk RNAseq, related to Figure 3

Table S2. DE genes of PIM TKD, scRNAseq, related to Figure 4

Table S3. siRNAs, LNA and Taqman primer sequences, related to STAR Methods

Document S2. Article plus supplemental information
