# Supplementary material for: PIM kinases regulate early human Th17 cell differentiation
Source: Cell Rep. 2023 Nov 30;42(12):113469. doi: 10.1016/j.celrep.2023.113469 (PMC10765319; doi:10.1016/j.celrep.2023.113469)
Supplement: Document S1. Figures S1–S8 [file mmc1.pdf]

Cell Reports, Volume *42*

Supplemental information

PIM kinases regulate early human Th17 cell differentiation

Tanja Buchacher, Ankitha Shetty, Saara A. Koskela, Johannes Smolander, Riina Kaukonen, António G.G. Sousa, Sini Junttila, Asta Laiho, Olof Rundquist, Tapio Lönnberg, Alexander Marson, Omid Rasool, Laura L. Elo, and Riitta Lahesmaa

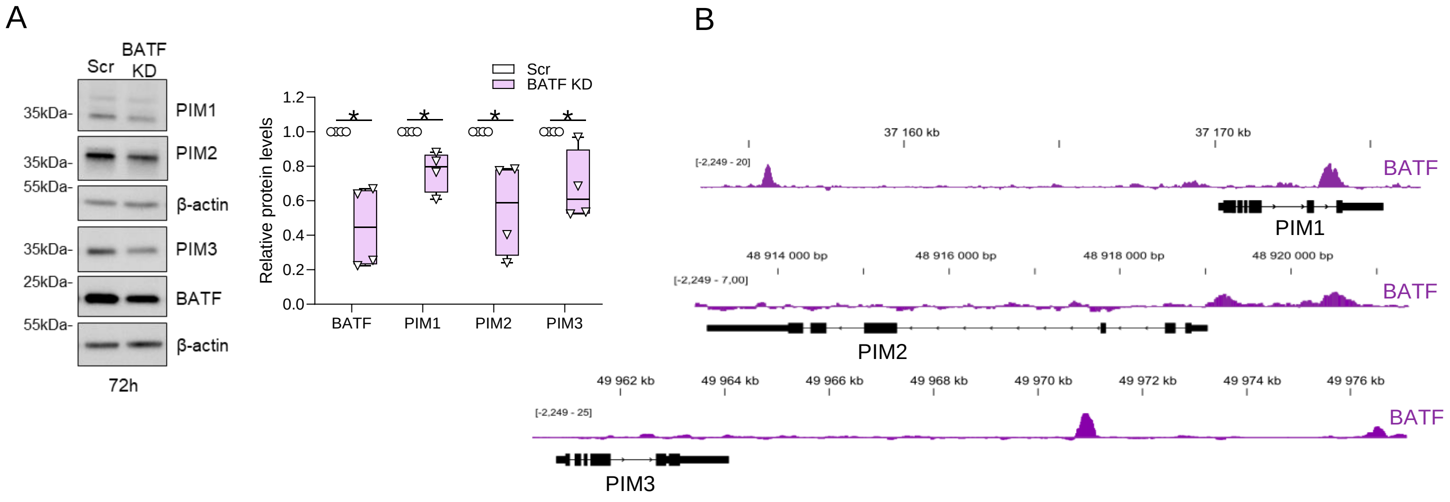

Figure S1. BATF silencing in human Th17 cells, Related to Figure 1. (**A**) Representative immunoblots show BATF, PIM1, PIM2 and PIM3 protein levels in Scr versus BATF KD cells at 72 h of Th17 differentiation. β-actin was used as loading control. Band intensities of target proteins were normalized to the loading control and relative to Scr control. Boxplots represent median and interquartile range, and whiskers extend to maximum and minimum values from four biological replicates (right panel). Statistical significance is calculated using two-tailed Student's t test (*p < 0.05). (**B**) IGV snapshots indicate the binding of BATF over intergenic regions of PIM1 (above panel) and PIM2 (middle) but not PIM3 (below panel) in Th17 cells cultured for 72 h. Figures were derived using bigwig files of BATF ChIP-seq data from our published study (Shetty A et al 2022; GEO: GSE174810).

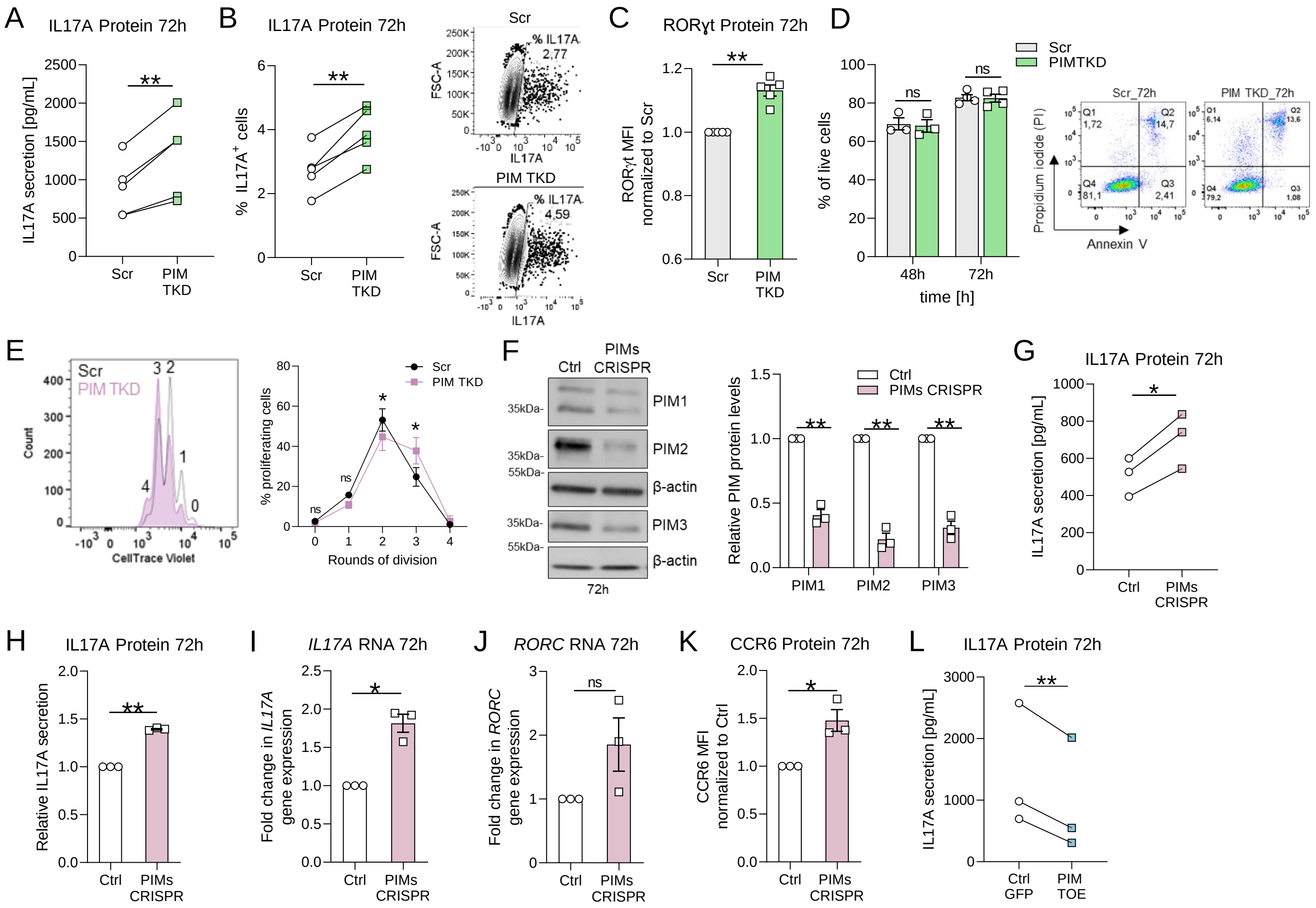

Figure S2. CRISPR-Cas9-mediated PIM ablation in human Th17 cells, Related to Figure 2. (**A**) Raw data of IL17A cytokine concentrations in the supernatants of PIM-deficient Th17 cells are shown at 72 h of polarization by ELISA from five biological replicates. (**B-C**) Intracellular staining of IL17A and RORɣt was assessed in Scr and PIM-deficient Th17 cells at 72 h of polarization by flow cytometry. The percent of IL17A positive cells (**B**) and the mean fluorescence intensity (MFI) values of RORɣt are shown from five biological replicates (**C**). (**D**) Cell viability of Scr control and PIM TKD Th17 cells was assessed at 48 and 72 h of differentiation by annexin V/propidium iodide (PI) staining using flow cytometry for four biological replicates. The percent of Annexin V and PI double-negative cells are depicted as live cells. (**E**) T cell proliferation of Scr control and PIM TKD Th17 cells was evaluated by flow cytometry in three biological replicates. Post-transfection, naive $CD4^{+}$ cells were stained with cell trace violet (CTV) and cultured for 96 h under Th17-polarizing conditions. The percent of CTV positive cells are shown. (**F-K**) PIM ablation in differentiating Th17 cells at 72 h using CRISPR Cas9 was confirmed by immunoblotting (**F**, left). Band intensities of PIM kinases from three biological replicates were normalized to β-actin and relative to Scr control (**F**, right). (**G**) Raw data of IL17A cytokine levels in supernatants of PIM CRISPR Th17 cells at 72 h of polarization are shown by ELISA from three biological replicates. (**H**) IL17A values were normalized for cell count by flow cytometry (live), and then normalized to Scr control. Data represent three biological replicates. (**I-K**) *IL17A* RNA levels (**I**), *RORC* RNA levels (**J**) and CCR6 surface expression (**K**) in PIM CRISPR Th17 cells at 72 h of polarization were analyzed in three biological replicates using qRT-PCR and flow cytometry analyses, respectively. (**L**) Raw data of IL17A cytokine concentrations in supernatants of PIM TOE Th17 cells are shown at 72 h of polarization by ELISA from three biological replicates. Graphs in **C**, **K** show mean fluorescence intensity values normalized to Scr and Ctrl control, respectively. Graphs in **I**, **J** depict FC normalized to the Scr control for three biological replicates. Plots (**A-L**) represent mean ± SEM. Statistical significance is calculated using two-tailed Student's t test (*p < 0.05, **p < 0.01, ns: not significant).

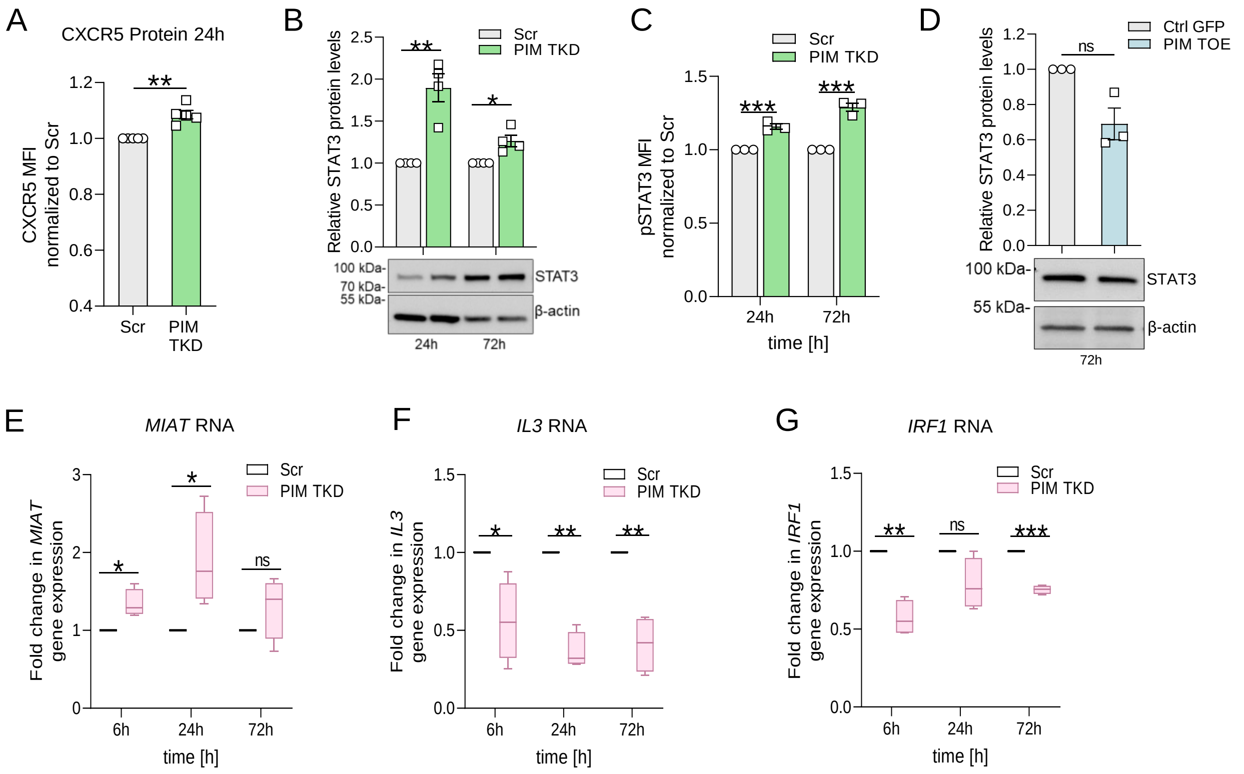

Figure S3. Validation of selected PIM RNA-seq targets, Related to Figure 3. (**A**) Surface-marker expression of CXCR5 was validated in PIM TKD Th17 cells at 24 h using flow cytometry. Bar plot shows mean fluorescence intensity (MFI) values normalized to Scr control for five biological replicates. (**B-D**) Upregulation of STAT3 protein levels was confirmed in PIM TKD cells at 24 and 72 h of polarization by immunoblotting. Band intensities of STAT3 protein were quantified in four biological replicates and normalized to β-actin and relative to Scr (**B**). (**C**) MFI of intracellular pSTAT3(Y705) was determined in 24 and 72 h PIM TKD Th17 cells by flow cytometry and normalized to Scr control for three biological replicates. (**D**) Overexpression of the three PIMs confirmed the downregulation of STAT3 protein by immunoblotting. Band intensities of PIMs from three biological replicates were normalized to β-actin and relative to GFP control. (**E-G**) RNA expression of *MIAT* (**E**), *IL3* (**F**) and *IRF1* (**G**) was analyzed in PIM-depleted Th17 cells over time, in four biological replicates by qRT-PCR. FC was normalized to Scr control. Boxplots represent median and interquartile range, and whiskers extend to maximum and minimum values. Plots (**A-D**) show mean ± SEM. Statistical significance is calculated using two-tailed Student's t test (*p < 0.05, **p < 0.01, ***p < 0.001, ns: non-significant).

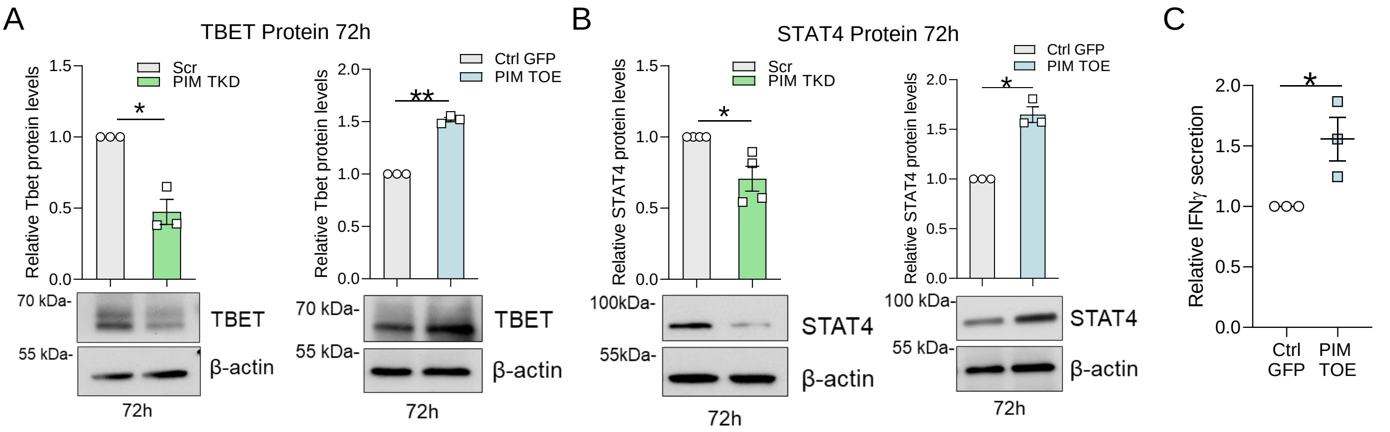

Figure S4. Protein expression of Th1-asscoaited factors in PIM TKD and/or PIM TOE in differentiating Th17 cells, Related to Figure 4. (**A-B**) Representative immunoblots show TBET (**A**) and STAT4 (**B**) protein levels in in PIM TKD (left panel) and PIM TOE (right panel) Th17 cells at 72 h in four and three biological replicates, respectively. β-actin was used as a loading control. Band intensities of TBET and STAT4 protein levels were normalized to loading control and relative to Scr or GFP control, respectively. (**C**) Secreted IFNɣ cytokine levels in supernatants of PIM TOE Th17 cells is shown at 72 h of polarization using ELISA for three biological replicates. Values were normalized for cell count by flow cytometry (live), and then normalized to Scr control. Plots (**A-C**) show mean ± SEM. Statistical significance is calculated using two-tailed Student's t test (*p < 0.05, **p < 0.01).

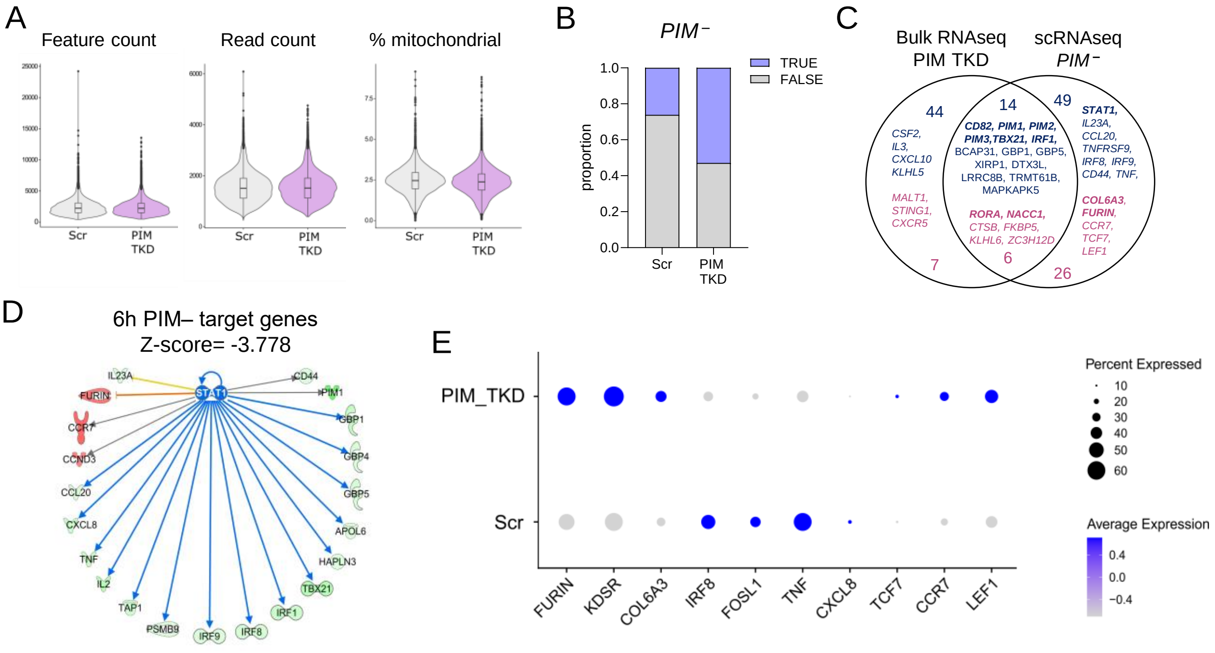

Figure S5. scRNA-seq of PIM-deficient Th17 cells at 6 h of differentiation, Related to Figure 4 (**A**) Quality control violin plots with boxplots and median of scRNA-seq dataset showing feature count distributions, UMI counts and the percent of mitochondrial genes for Scr and PIM TKD. (**B**) Upon scRNA-seq analysis, the proportion of PIM negative (*PIM–*) cells in blue are shown in Scr (control) versus PIM TKD Th17 cells at 6 h for one biological replicate. (**C**) Differential expression analysis of scRNAseq data was performed on Scr (total) versus *PIM⁻* cells in PIM TKD samples at 6 h of Th17 cell differentiation (FDR < 0.05, log2FC > 0.24). Venn diagram shows the overlay of DE genes between the bulk and scRNA-seq data. (**D**) Based on scRNA-seq data, *STAT1* was predicted as an upstream regulator of the PIM DE genes using the IPA "upstream regulator" prediction tool (Z score < −3.778). Blue and red lines represent positively and negatively regulated genes, respectively. Grey arrows stand for effect not predicted). (**E**) The expression of selected differentially expressed genes in Scr (control) and *PIM⁻* in PIM TKD samples are shown at 6 h of differentiation. Scaled scRNA-seq dot plot depicting the DE genes of interest on the x axis. The color scale represents the average expression of a given gene in the cluster, and the size of the dot represents the percent of cells that express a given gene.

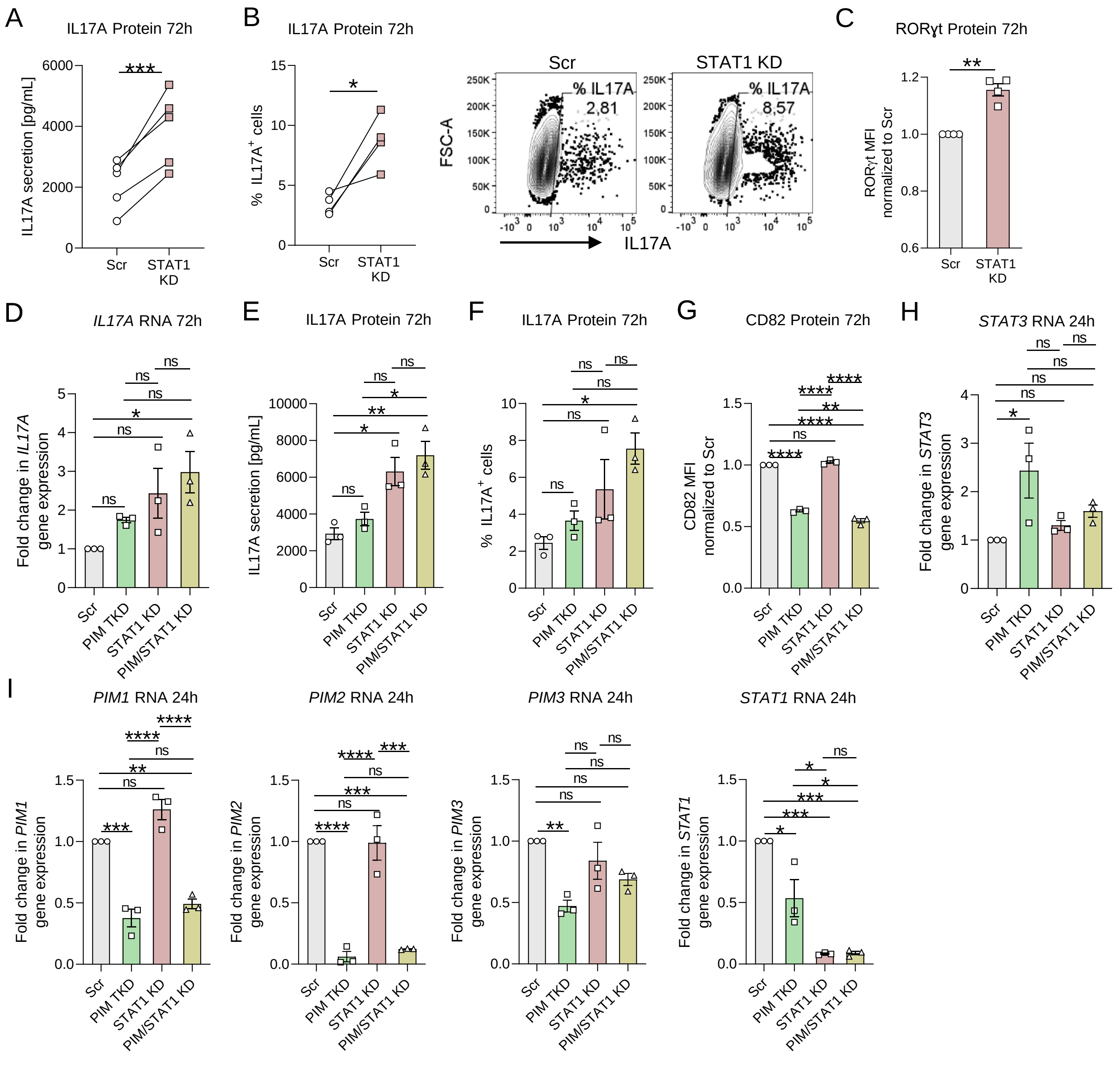

Figure S6, STATI single- and STAT1-PIM double-KD during early human Th17 differentiation, Related to Figure 5. (**A**) Raw data of IL17A cytokine concentrations in supernatants of STAT1 KD at 72 h of polarization are shown by ELISA from five biological replicates. (**B-C**) Intracellular staining of IL17A and RORγt was assessed in Scr and STAT1-deficient Th17 cells at 72h of polarization by flow cytometry. The percent of IL17A positive cells (**B**) and the mean fluorescence intensity (MFI) values of RORγt (**C**) were analyzed in four biological replicates. (**D-I**) Single- and double-knockdown (KD) of STAT1 and PIMs in Th17 cells are shown from three biological replicates. (**D-F**) *IL17A* RNA levels (**D**), raw data of secreted IL17A levels (**E**) and intracellular staining of IL17A (**F**) were analyzed in STAT1 and PIM single- and double-deficient Th17 cells at 72 h of polarization using qRT-PCR, ELISA and flow cytometry, respectively. (**G**) CD82 surface expression in PIM and/or STAT1 KD Th17 cells at 72 h of differentiation was analyzed by flow cytometry. (**H-I**) RNA expression of *STAT3* (**H**), *PIMs* and *STAT1* (**I**) was analyzed in PIM-depleted Th17 cells at 24 h of differentiation using qRT-PCR. Graphs in **C** and **G** show mean fluorescence intensity values normalized to Scr control. Graphs in **D**, **H**, and **I** depict FC normalized to the Scr control. Plots (**A-I**) show mean ± SEM. Statistical significance in graphs **A-C** was calculated using two-tailed Student's t test. Statistical significance in graphs **D-I** was calculated using the one-way ANOVA, Tukey's multiple comparisons test (*p < 0.05; **p < 0.01; ***p < 0.001; ****p < 0.0001, ns: not significant).

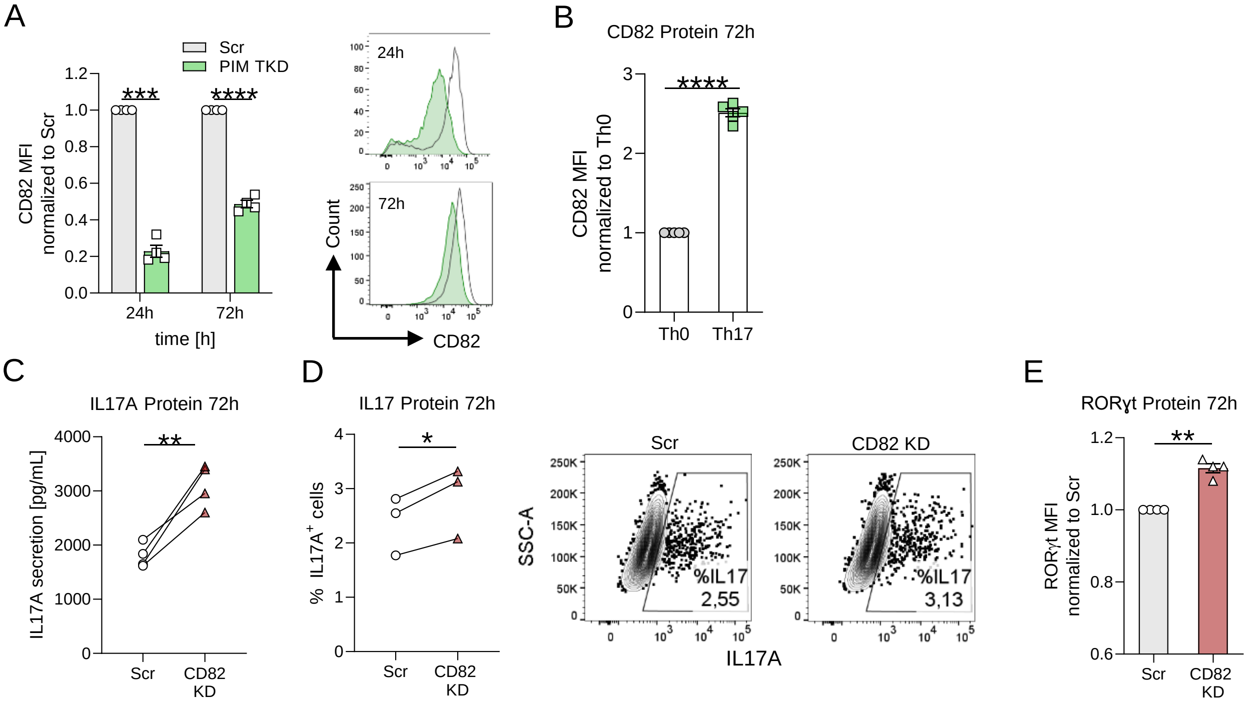

Figure S7. CD82 silencing during human Th17 cell differentiation, Related to Figure 5. (**A**) Surface-marker expression of CD82 was validated in PIM TKD Th17 cells at 24 and 72 h of differentiation using flow cytometry. Bar plot shows MFI values normalized to Scr control, for four biological replicates (left panel). Representative histograms are shown on the right. (**B**) Flow cytometry results revealed the expression of CD82 in naive $CD4^+$ T cells cultured for 72 h under conditions of activation (Th0) and Th17 differentiation. Graph shows MFI values normalized to Th0, for five biological replicates. Statistical significance was calculated by comparing each condition to Th0. (**C**) Raw data of IL17A cytokine concentrations in supernatants of CD82 KD Th17 cells at 72 h of polarization are shown by ELISA from four biological replicates. (**D-E**) Intracellular staining of IL17A and RORɣt was assessed in Scr and CD82-deficient Th17 cells at 72 h of polarization by flow cytometry. The percent of IL17A positive cells (**D**) and MFI values of RORɣt (**E**) are shown for three and four biological replicates, respectively. MFI values are normalized to Scr control. Plots (**A-E**) show mean ± SEM. Statistical significance was calculated using two-tailed Student’s t test (*p < 0.05; **p < 0.01; ***p < 0.001, ****p < 0.0001).

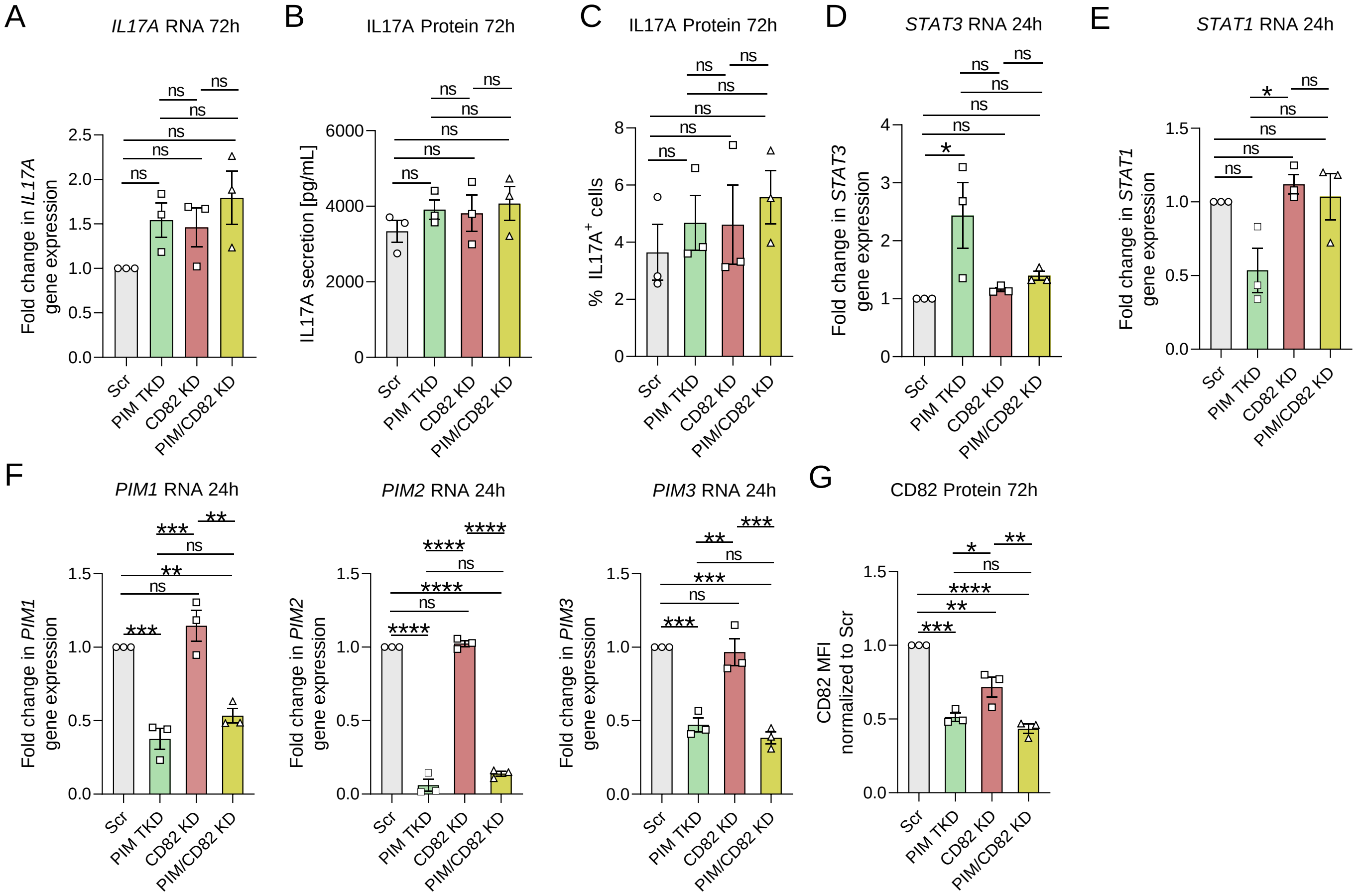

Figure S8. CD82 single- and CD82-PIM double-KD during early human Th17 differentiation, Related to Figure 5. (**A-G**) Single- and double-knockdown (KD) of CD82 and PIMs in Th17 cells are shown from three biological replicates. (**A-C**) *IL17A* RNA levels (**A**), raw data of secreted IL17A levels (**B**) and intracellular staining of IL17A (**C**) were analyzed in CD82 and PIM single- and double-deficient Th17 cells at 72 h of polarization using qRT-PCR, ELISA and flow cytometry, respectively. (**G**) CD82 surface expression in PIM and/or CD82 KD Th17 cells at 72 h of differentiation was analyzed by flow cytometry. (**D-F**) RNA expression of *STAT3* (**D**), *STAT1* (**E**) and *PIMs* (**F**) was analyzed in PIM-depleted Th17 cells at 24 h of differentiation using qRT-PCR. Graph **G** shows MFI values normalized to Scr control. Graphs in **A**, and **E-F** depict FC normalized to the Scr control. Plots (**A-G**) show mean ± SEM. Statistical significance was calculated using the one-way ANOVA, Tukey's multiple comparisons test (*p < 0.05; **p < 0.01; ***p < 0.001; ****p < 0.0001, ns: not significant).
